# The isolation and characterization of renal cancer initiating cells from human Wilms' tumour xenografts unveils new therapeutic targets†

**DOI:** 10.1002/emmm.201201516

**Published:** 2012-12-13

**Authors:** Naomi Pode-Shakked, Rachel Shukrun, Michal Mark-Danieli, Peter Tsvetkov, Sarit Bahar, Sara Pri-Chen, Ronald S Goldstein, Eithan Rom-Gross, Yoram Mor, Edward Fridman, Karen Meir, Amos Simon, Marcus Magister, Naftali Kaminski, Victor S Goldmacher, Orit Harari-Steinberg, Benjamin Dekel

**Affiliations:** 1 Pediatric Stem Cell Research Institute, Edmond and LiliSafra Children's Hospital, Sheba Medical CenterRamat-Gan, Israel; 2 Sheba Centers for Regenerative Medicine and Cancer Research, Sheba Medical CenterRamat-Gan, Israel; 3 Sackler School of Medicine, Tel Aviv UniversityIsrael; 4 Department of Molecular Genetics, Weizmann Institute of ScienceRehovot, Israel; 5 The Maurice and Gabriela Goldschleger Eye Research Institute, Sheba Medical CenterRamat-Gan, Israel; 6 Mina and Everard Goodman Faculty of Life Sciences, Bar-Ilan UniversityGivaat-Shmuel, Israel; 7 Department of Pediatric Surgery, Hadassah Medical Center, Hebrew UniversityJerusalem, Israel; 8 Department of Urology, Sheba Medical CenterRamat-Gan, Israel; 9 Department of Pathology, Sheba Medical CenterRamat-Gan, Israel; 10 Department of Pathology, Hadassah Medical Center, Hebrew UniversityJerusalem, Israel; 11 University of Pittsburgh School of MedicinePittsburgh, PA, USA; 12 ImmunoGen, Inc.Waltham, MA, USA; 13 Division of Pediatric Nephrology, Edmond and Lili Safra Children's Hospital, Sheba Medical CenterRamat-Gan, Israel

**Keywords:** cancer initiating cells, cancer stem cells, kidney stem cells, renal progenitor cells, targeted therapy

## Abstract

There are considerable differences in tumour biology between adult and paediatric cancers. The existence of cancer initiating cells/cancer stem cells (CIC/CSC) in paediatric solid tumours is currently unclear. Here, we show the successful propagation of primary human Wilms' tumour (WT), a common paediatric renal malignancy, in immunodeficient mice, demonstrating the presence of a population of highly proliferative CIC/CSCs capable of serial xenograft initiation. Cell sorting and limiting dilution transplantation analysis of xenograft cells identified WT CSCs that harbour a primitive undifferentiated – NCAM1 expressing – “blastema” phenotype, including a capacity to expand and differentiate into the mature renal-like cell types observed in the primary tumour. WT CSCs, which can be further enriched by aldehyde dehydrogenase activity, overexpressed renal stemness and genes linked to poor patient prognosis, showed preferential protein expression of phosphorylated PKB/Akt and strong reduction of the miR-200 family. Complete eradication of WT in multiple xenograft models was achieved with a human NCAM antibody drug conjugate. The existence of CIC/CSCs in WT provides new therapeutic targets.

→See accompanying article http://dx.doi.org/10.1002/emmm.201202173

## INTRODUCTION

Recent years have witnessed the exciting discoveries of cancer initiating cells/cancer stem cells (CIC/CSCs) in solid tumours, including those of the breast and the brain (Al-Hajj et al, 2003; Singh et al, 2003). By applying principles derived from stem cell research, human CIC/CSCs are functionally defined by their enhanced capacity to regenerate cancers using xenograft mouse models. Similar to normal stem cells, CIC/CSCs are capable of self-renewal, which can be studied in serial transplantation assays. Additionally, cancers derived from purified CIC/CSCs recapitulate the heterogeneous phenotypes of the parental cancer from which they were derived, reflecting the differentiation capacity of CSCs (Reya et al, 2001; Singh et al, 2004). These observations suggest that CIC/CSCs contain the complete genetic programs necessary to initiate and sustain tumour growth. Additionally, the persistence of these cancer stem/progenitor cells after standard treatments has been put forward as an explanation for recurrence of tumours (Clarke et al, 2006; Reya et al, 2001). Much of the more recent research on CIC/CSCs has focused on adult cancers derived from highly differentiated tissues (Al-Hajj et al, 2003; Collins et al, 2005; Ginestier et al, 2007; O'Brien et al, 2007; Schatton et al, 2008).

Paediatric solid tumours are a group of childhood cancers that recapitulate embryonic/foetal development and are mainly embryonic in origin. Given the considerable differences in tumour biology between adult and paediatric solid cancers the initial question is whether or not CIC/CSCs exist and if so, whether there may be significant differences in their presence and behaviour compared to adult counterparts (Gillespie, 2011). Identification of such cells may promote targeted therapies for paediatric solid tumours.

Wilms' tumour (WT) is the most frequent tumour of the genitourinary tract in children and serves as a prototype for studying the relation between development and tumourigenesis (Rivera & Haber, 2005). Based on its tri-phasic histology [that of cell lineages similar to those observed during kidney development; undifferentiated blastema along with stromal and epithelial derivatives (such as immature tubules and glumeruloid bodies)], WT is hypothesized to harbour CIC/CSCs with the capacity for self-renewal and for abnormal and disorganized differentiation along developmental pathways that occur during nephrogenesis. Nevertheless, the cancer stem cell model has yet been applied for WT.

Preliminary evidence for the existence of a tumour stem/progenitor population in classical tri-phasic human WT comes from our *in vitro* work with low-passage WT cultures derived from primary tumours showing after immunophenotyping that a sorted NCAM^+^ cell fraction is highly clonogenic and enriching for a renal stemness signature set predicted by previous WT microarray experiments (Dekel et al, 2006; Metsuyanim et al, 2008; Pode-Shakked et al, 2009). In contrast, sorting according to CD133, a marker proposed to identify embryonic renal progenitors and paediatric CICs (Gillespie, 2011; Pode-Shakked et al, 2009; Ronconi et al, 2009), failed to generate a similar cell phenotype. Nevertheless, *in vivo* assays, *e.g.* serial cell transplantation experiments with animal models are the gold standard for identifying CIC/CSCs (Clarke et al, 2006). In this regard, WT cells, as opposed to surgical tissue samples of WT, are known for their inability to generate tumour xenografts (Xn) limiting the application of functional definitions of CIC/CSCs to human WT (Wen et al, 1997). Establishment of WT Xns from cell suspensions of fresh primary WT has been estimated at 30% graft take (Wen et al, 1997) and in our experience approximately 10%, while after culture and *in vitro* growth of WT cells, Xn formation is unattainable, making long-term serial transplantation assays with human cells impossible. Furthermore, tumourigenic favourable histology WT cell lines are not available. Therefore, taking into account these inherent limitations of WT and the fact that cells within WT, as with other paediatric solid tumours, are less accessible (compared to adult carcinomas), model systems that allow for *in vivo* studies of WT at the single cell level are warranted.

Herein, we have propagated human WT *in vivo*, demonstrating the presence of a population of highly proliferative CIC/CSCs capable of serial xenograft initiation and propagation. This has allowed, for the first time, a robust definition of the CIC/CSC phenotype leading to discovery of therapeutic targets for WT eradication. Molecular analysis of CIC/CSCs in WT Xn revealed distinct genes, signaling elements and miRNAs likely relevant to their function as undifferentiated “blastema” cells. The generation of xenografts carrying cell-repopulating human developmental malignancies with aggressive phenotypes may serve as a more reliable platform compared to cell-line-based xenografts or *in vitro* propagation for the discovery of CICs and future targeted therapies.

## RESULTS

### WT xenografts can be established from surgical tissue samples but not from primary WT cells

To study human WT initiating activity, we injected primary WT cells into NOD/SCID immunodeficient recipient mice. Primary WT cells were obtained from a cohort of tri-phasic favourable histology WT lacking mutations in β-catenin, WT1 or WTX, which represent genetic alternations in a subset of WT (Maiti et al, 2000; Rivera et al, 2007). Following digestion of surgical samples of WT and injection of a single cell suspension (up to 2 × 10^7^ cells) we found an extremely low frequency of xenograft formation: 2/20 mice injected with tumour cells from four different WT sources developed tumours 6 months after transplantation. These numbers are in line with previous reports (Wen et al, 1997). Furthermore, attempts to use primary WT cell cultures confirmed the inability of cultured cells (as low as P1, with up to 10^8^ cells injected) to initiate tumour Xns in mice (*n* = 0/25 from five WT patients, analysed up to a year after injection). In contrast, primary Wilms' tumour fragments (2 × 2-mm-minced pieces) derived from 10 different WT patients resulted in robust Xn formation upon grafting; graft take was observed for 8 out of the 10 WT sources within 2–6 months (altogether, Xns were established in 40/50 mice). We therefore used these xenograft models containing primary human WT to determine the existence of CIC/CSCs. Xenografts are critical for this study because of the difficulty to routinely obtain paediatric primary solid tumours, which are less frequent than adult cancers.

### Human WT initiation and propagation *in vivo* via highly proliferating tumour cells

Having established 1st generation Xns from tissue samples obtained from eight different WT sources, we then analysed whether these Xns could be initiated and propagated following dissociation of Xn tissue and injection of the derived single cell suspension. Interestingly, we found that 1st generation Xns established from four WT sources (W011, W013, W014 and W016) could be readily initiated and propagated in mice through the injection of 0.5–1 × 10^6^ Xn derived cells. Lower cell numbers (10^4^ cells) were mostly sufficient to initiate later generation xenografts (>4th generation). Overall, these Xns, termed propagatable WT Xns (p-WT Xn) showed increased WT-initiating activity, serving as a WT reservoir for the study of tumour-initiating cells. The experimental outline for Xn generation is depicted in Supporting Information Fig S1.

**Figure 1:**
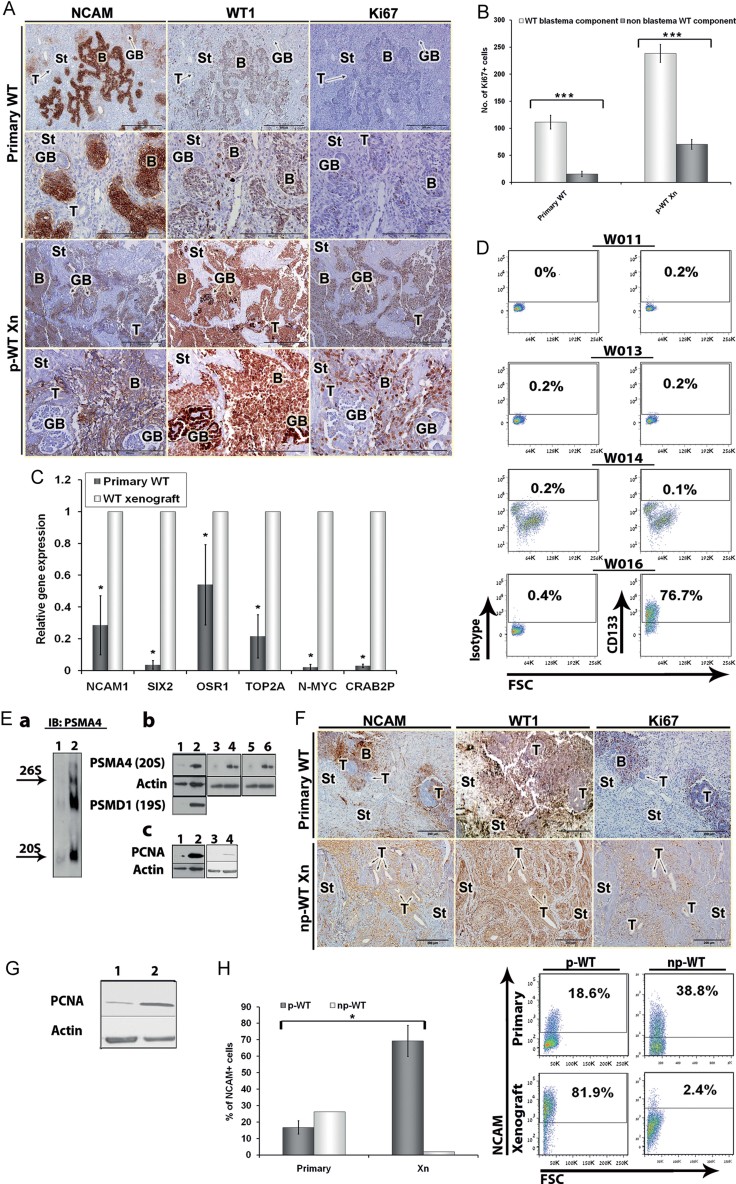
p-WT Xns are enriched for highly proliferating tumour cells capable of serial Xn initiation while np-WT Xn lack these characteristics Immunohistochemical staining presented in serial sections for NCAM, WT1 and Ki67 in a representative primary tumour (W013) and its derived propagatable Xn (p-WT Xn). Staining demonstrates recapitulation of the original tumour by the WT Xn with an increase in the number of cells positive for NCAM and Ki67, markers of undifferentiated blastema and cell proliferation, respectively. WT1, the diagnostic pathologic marker of WT, is expressed in both primary and Xn tumours on the same structures (mainly blastema). (Scale bars = 500 µm and 200 µm for magnifications 10× and 40×, respectively).Quantitative comparison of proliferating cells (Ki67+) between WT blastema and non-blastema components in primary WTs and their derived Xn (W013, W014 and W013 Xn, W014 Xn). The number of Ki67+ cells was significantly higher in WT blastema in both primary WTs and WT Xn. (***p = 1.6025E−05 and 3.82489E−07 respectively).qRT-PCR analysis of renal progenitor (*i.e. SIX2, OSR1*) and poor prognostic (*i.e. TOP2A*, *N-MYC, CRAB2P*) genes compering between primary WTs (*n* = 3) and their derived WT Xn (*n* = 3), demonstrates significantly elevated mRNA levels of these genes in WT Xn (*p = 0.031). All values were normalized to WT Xn (therefore = 1). Results are presented as the mean ± SEM of five separate experiments.Flow cytometric analyses for CD133 expression and corresponding isotype controls in 4 WT Xn showing negligible levels in 3 of the 4 Xn (W011, W013 and WO14) while the 4th (WO16) show high percentages of cells expressing CD133.Proteasomal levels were examined in primary tumours (*1-W014*, *3-W019, 5*-*W020*) compared to their derived Xenografts (*2- W014 Xn, 4- W019 Xn, 6- W020 Xn*). (a) Proteasomal complexes were analysed by nondenaturing page analysis. (b) Total proteasomal subunit protein levels by immunoblot analysis of polyacrylamide-SDS gel. (c) PCNA levels were analysed by immunoblot in primary tumours *versus* their derived Xn, showing an increase in PCNA levels in WT Xn.Immunohistochemical staining presented in serial sections for NCAM, WT1 and Ki67 in a representative primary tumour (W017) and its derived non-propagatable Xn (np-WT Xn) demonstrating decreased expression of NCAM, WT1 and Ki67 along with dramatic reduction in the undifferentiated blastema component in the latter; (Scales bars = 200 µm, Magnification 20×).Immunobloting analysis for the proliferation marker PCNA showing higher levels in p-WT Xn (2) compared to np-WT Xn (1).Average percentage of NCAM expressing cells in tumour sources and their derived Xn showing an increase in cells expressing NCAM in p-WT Xn and a decrease in np-WT Xn compared to their primary WT source (Left panel). Representative FACS plots showing increased NCAM levels in W016 Xn (p-WT Xn) in comparison to its tumour source (primary W016) while W017 Xn (np-WT Xn) shows the opposite (Right panel). In all comparison experiments *n* = 2 for each tumour type and three mice were used for each tumour source (total of 12 mice).

Having observed that p-WT Xn harbour enhanced WT-initiating activity, we next determined their characteristics in comparison with parental tumours. Histological examination of p-WT Xn (W013) revealed all WT tri-phasic components (blastema, stroma, epithelium) but with enriched blastema compared to parental tumour, indicating the generation of a blastema-predominant WT. Interestingly, patients with recurrent blastema-predominant WT have been classified at very high risk with low survival rates (Reinhard et al, 2008). We performed immunostaining of p-WT Xn and parental WT for the following: WT1, a specific clinical diagnostic marker for WT; NCAM1, a putative marker for a tumour progenitor population previously shown in *in vitro* assays of primary cell cultures of WT (Pode-Shakked et al, 2009); and Ki-67, a cell proliferation marker. We observed NCAM1 and Ki-67 expression in p-WT Xn, mostly within the expanded blastema component (Fig 1A). Quantification of proliferating cells (Ki67^+^) in WT blastema and non-blastema components revealed significantly higher numbers of Ki-67^+^ cells in the blastema. This observation was repeated in both primary WTs and their derived Xn (Fig 1B).

We further interrogated p-WT Xn at both gene and protein levels. qRT-PCR analysis for human transcript levels showed significant elevation of renal “stemness” and progenitor genes (Dekel et al, 2006) that mark the early renal lineage, including *SIX2* and *OSR1,* compared to their primary tumour of origin. Importantly, both *SIX2* and *OSR1* have been shown to identify self-renewing epithelial stem/progenitor cells in the metanephric mesenchyme of the developing kidney (Kobayashi et al, 2008; Mugford et al, 2008; Self et al, 2006). OSR1^+^ cells have been shown to possess multipotency as they are able to differentiate into all lineages of all metanephric kidney components in early development (Mugford et al, 2008). In addition, p-WT Xn overexpressed genes known to associate with poor prognosis WT: *TOP2A, N-MYC* and *CRAB2P*, correlating with a highly aggressive tumour phenotype (i.e blastema predominance; Green et al, 1994; Takahashi et al, 2002; Wittmann et al, 2008; Fig 1C). FACS analysis for NCAM and CD133 levels in cells derived from p-WT Xn and from parental WT revealed significantly higher NCAM levels in p-WT Xn (50–90%) while CD133 expression was completely absent in three out of the four p-WT Xn (*n* = 12 mice; Fig 1D). This elevation in NCAM levels was observed in comparison with both P0 and P2-4 cultures of primary (parental) WT cells (Table 1, for patient and tumour characteristics see Supporting Information Table S1). Interestingly, the one p-WT Xn with elevated CD133 levels could be propagated only when large numbers of cells (>5 × 10^6^) were continually used. In contrast, 100-fold fewer CD-133 negative cells were required to propagate and sustain late generation Xns of p-WT. Finally, proteasome and proliferating cell nuclear antigen (PCNA) protein levels increased in p-WT Xn compared to parental tumour tissue (Fig 1E). Elevated proteasome and PCNA levels had been previously shown to be associated with increased cell proliferation and in glioblastoma CIC/CSCs (Monticone et al, 2009; Wang et al, 1998).

**Table 1:** Expression of NCAM and CD133 in various primary Wilms' tumours (WT) and in respective fresh or cultured xenograft derived cells

Patient code	Passage	Total NCAM	Total CD133	Passage	Total NCAM	Total CD133			
Primary WT									
W002	0	33.75	11.3	2–4	32.9 ± 14	34.6 ± 19.2				
W003	0	6.8	0.2	2–4	6 ± 1.9	1 ± 0.1				
W004	0	39.7	8.5	2–4	38 ± 13.7	38.7 ± 1.9				
W005	0	24.8	22.8	2–4	20.7 ± 12.9	32 ± 32.8				
W006	0	10.9	0.7	2–4	18.3 ± 4.9	4.1 ± 1.5				
W007	0	33.9	52.1	2–4	23.5 ± 5.9	72.9 ± 11.2				
W009	0	34.7	0.4	2–4	50.7 ± 18.8	2.2 ± 0.9				
W010	0	39.5	7.1	2–4	33.6 ± 13.3	74.3 ± 1.3				
W011	0	ND	ND	2–4	ND	ND				
W013	0	9.8	ND	2–4	ND	ND				
W014	0	22.6	ND	2–4	ND	ND				
W016	0	18.6	11.3	2–4	36.8 ± 2.3	20.7 ± 20.1				
W017	0	38.7	ND	2–4	ND	ND				

Tumour source	Passage	Total NCAM	Total CD133	Total PSA-NCAM	Total CD44	ALDH1	Passage	Total NCAM1	Total CD133
WT xenografts									
W011	0	90.9 ± 6.6	0.3 ± 0.4	39.9 ± 30.6	22.9 ± 12.2	4.3 ± 2.3	2–4	84.8 ± 8.6	18.1 ± 5.3
W013	0	72 ± 47.2	0.1 ± 0.2	9.3 ± 4.3	22.7 ± 6.7	0.5	2–4	16.2	1.3
W014	0	84.2 ± 24.9	0.2 ± 0.3	26.8	10.3 ± 7.4	4.7 ± 0.9	2–4	17.5 ± 8.2	65.4
W016	0	51.9 ± 39.7	75.9	6.3 ± 3.2	16.2	12.3 ± 2.2	2–4	10.6	15.6
W017	0	1.9	0.85	1.27	12.2	ND	2–4	ND	ND

Thus, increased WT-initiating activity of p-WT Xn correlated with blastema-predominant, highly proliferating tumours that are enriched for markers of renal stem/progenitor cells. In contrast, the 1st generation Xns that formed via tissue grafting but did not propagate in mice following dissociation and single cell injections (termed np-WT Xn), showed an opposite phenotype: np-WT Xn did not expand the blastema and featured decreased NCAM, WT1 and Ki67 immunostaining (Fig 1F) and lower expression levels of the proliferation marker PCNA (Fig 1G). FACS analysis of np-WT Xn revealed lower NCAM levels compared to parental tumour sources (Fig 1H). In fact, the high NCAM, low CD133 levels observed on FACS for tumourigenic p-WT Xn were absent from np-WT Xn as well as from non-tumourigenic primary P0 or P2-4 WT cultures (Fig 1H and Table 1).

All subsequent experiments were performed on p-WT Xn.

### *In vitro* growth conditions modulate WT-initiating activity

Since we observed that culture of WT cells derived from primary tumours abrogates their tumour-initiating capacity, we determined the effects of short-term culture of p-WT Xn cells as monolayer or spheres on *in vivo* WT formation (Supporting Information Figs S1, S2 and Tables S2 and S3). Fresh p-WT Xn cells injected to NOD-SCID and SCID-Biege mice, generated tumours recapitulating the histologic elements found in their parental tumour source. Concurrently, FACS analysis of cultured p-WT Xn cells (P2–P4) showed decreased NCAM levels (Table 1) suggesting depletion of the stem-like component in the tumour, further supporting the use of fresh p-WT Xn cells for dissection of WT-initiating activity (Table 2).

**Table 2:** Qualitative characteristics of primary WT and WT Xn

	Parent tumour	p-WT Xn	np-WT Xn
Histology	Tri-phasic	Tri-phasic, expanded blastema	Tri-phasic, blastema reduced, expended epithelia and stroma
Surface markers	Low/mid levels of NCAM and CD133	High levels of NCAM reduced levels of CD133	Reduced levels of NCAM
Proliferation markers	Low levels of KI-67/PCNA/proteosome	Elevated KI-67/PCNA/proteosome	Low levels of KI-67/PCNA/proteosome
Embryonic renal stem cell markers	Low/mid levels	Elevated	–

### The NCAM^+^ population contains the WT Xn initiating and propagating cells

**Figure 2:**
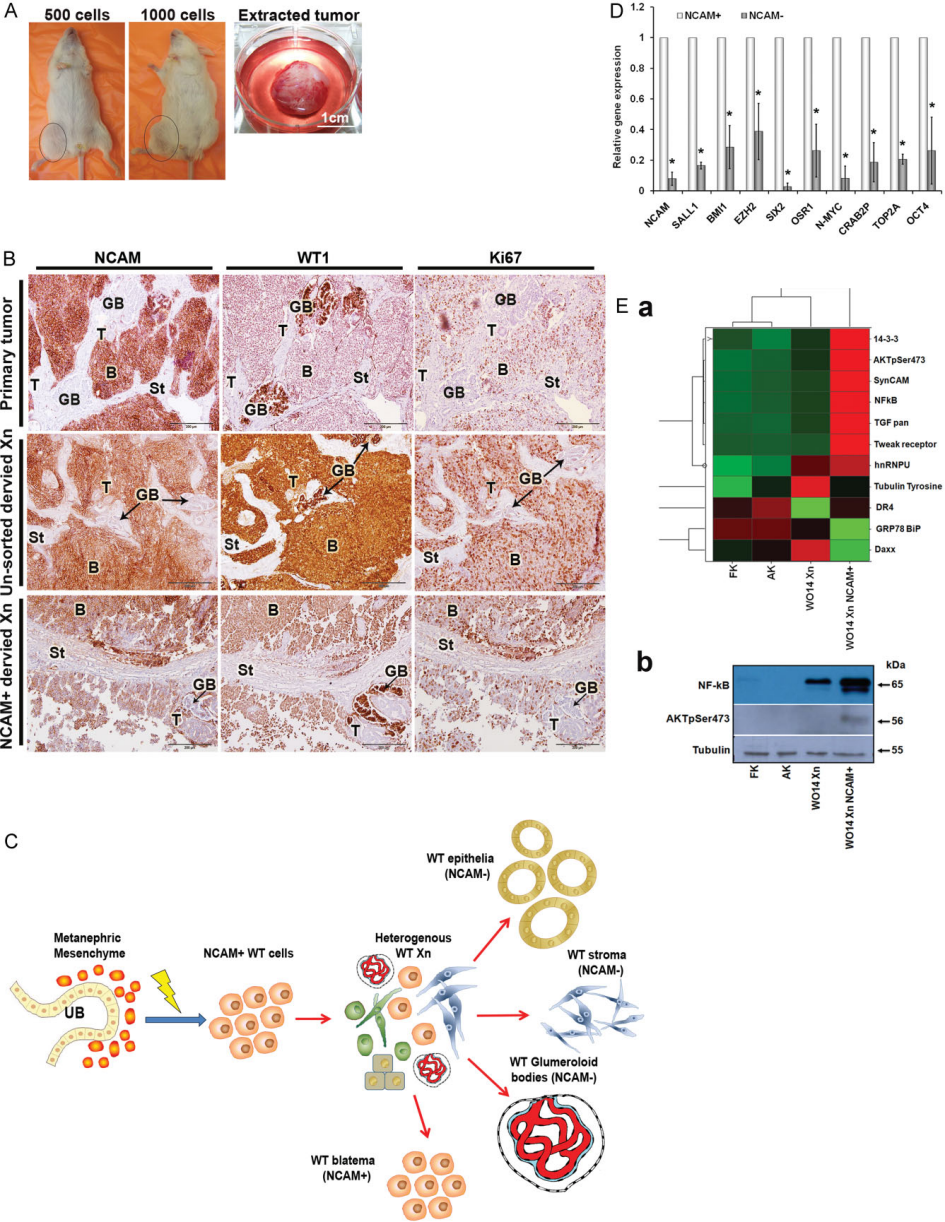
WT initiating and propagating cells are contained within the NCAM^+^ population Representative images of NOD-SCID mice injected with 1000 and 500 NCAM^+^ cells that developed tumours and of a tumour extracted from a mouse injected with NCAM^+^ cells showing encapsulation of the tumour mass that separate it from host tissues (Scales bars = 100 µm, magnification ×10 and ×20).Immunohistochemical staining presented in serial sections for NCAM, WT1 and Ki67 in a representative primary tumour and its derived xenografts generated from unsorted cells or from NCAM^+^ cells showing recapitulation of the original tumour. The NCAM^+^ cells formed tumours that contain both undifferentiated NCAM^+^ blastemal structures and well-differentiated NCAM^−^ tubular structures. WT1, the marker used in clinical practice for histopathoplogic diagnosis of WT, is abundantly expressed in both WT xenografts on similar structures as in the primary tumour. Ki67 is more abundant in the WT Xn than in their primary source. T-Tubules; B-Blastema; GB-Glomeruloid bodies; St-Stroma. (Scales bars = 200 µm; Magnification 20×).A scheme illustrating the putative derivation of NCAM^+^ WT cells from the transformed MM of the human foetal kidney and their capability to initiate p-WT xenografts that recapitulate the histology of their parental tumour, consisting of NCAM^+^ blastema cells (possibly formed by self renewal) as well as the differentiated features seen in primary human WTs (immature tubular epithelia, stroma and glomeruloid bodies).qRT-PCR analysis for the expression of renal progenitor (*i.e. NCAM, SALL1, SIX2, OSR1*), stemness (*i.e. BMI1, EZH2, OCT4*) and poor prognostic (*i.e. TOP2A, N-MYC, CRAB2P*) genes between NCAM^+^ and NCAM^−^ WT Xn cells (*n* = 3), demonstrate significantly elevated mRNA levels of these genes in NCAM^+^ compared to NCAM^−^ cells (*p* = 0.031). The values for the NCAM^+^ cells were used to normalize (therefore = 1) and all other values were calculated with respect to them. Results are presented as the mean ± SEM of five separate experiments.Increased expression of TWEAK-R/TGFb/PKBpSer473/14-3-3/NF-κB specifically in WT xenografts derived from NCAM^+^ cell. (a) Heat map of Panorama® Antibody Array illustrating expression of differentially expressed proteins in early WT xenograft generated from NCAM^+^ cells (WO14 Xn NCAM^+^), WT xenograft generated from cells that were not separated (W014 Xn), human adult kidney (AK) and human foetal kidney (hFK) tissues; (b) Western Blot analysis of NF-kB and AKTpSer473.

Human CICs can be functionally identified by immunosorting according to surface markers followed by transplantation in mice in limiting dilutions (Al-Hajj et al, 2003; Clarke et al, 2006; Lapidot et al, 1994; Pode-Shakked et al, 2009). Taking into account our previous findings (Pode-Shakked et al, 2009) and the results described above, we reasoned that NCAM^+^ cells may play a role in WT Xn initiation and propagation. We therefore sorted p-WT Xn cells into NCAM-positive and NCAM-negative cell populations (Fig S3B). We then injected serial dilutions of pure cell fractions into the right (NCAM^+^) and left (NCAM^−^) flanks of NOD/SCID mice. Strikingly, only the NCAM^+^ fraction was able to form tumours in NOD/SCID mice (Fig 2A). Moreover, as few as 500 NCAM^+^ cells were sufficient to initiate tumours, in comparison with >10^4^ unsorted p-WT Xn cells required for Xn initiation (Table 3). The tumours generated from NCAM^+^ cells could be further propagated upon serial transplantation in NOD/SCID mice (currently generation 5), while histology and immunostaining of these tumours disclosed a blastema compartment that expressed NCAM, but also NCAM^−^ differentiated epithelial-like structures and glumeruloid bodies, recapitulating the histology of both the parental WT and the p-WT Xn from which they were derived (Fig 2B). Thus, the NCAM^+^ cell fraction is enriched for WT-initiating activity and also contains CSC properties of differentiation and self-renewal (Clarke et al, 2006; Reya et al, 2001). Characterization of this fraction by qRT-PCR showed significant elevation of early renal progenitor transcripts (*SIX2, OSR1, SALL1*), stemness factors (*BMI1, EZH2, OCT4*) and WT poor prognostic factors (*TOP2A, N-MYC, CRAB2P*) compared to NCAM^−^ cells (Fig 2D). Furthermore, the enriched WT-initiating activity of NCAM^+^ cells might be related to specific biological pathways. We therefore performed a panorama antibody array (725 validated antibodies that detect proteins associated with a variety of key cell signaling and gene regulation pathways) on extracts of p-WT Xns early after initiation by sorted NCAM^+^ cells, un-sorted p-WT Xn and normal kidneys (Supporting Information Table S4). We considered differentially expressed proteins only when at least a fivefold change appeared among samples. For most proteins, there was little difference in expression; nevertheless, we detected a small subset of proteins preferentially activated in NCAM^+^ derived tumours and confirmed by Western blot (Fig 2E). Strikingly, in addition to the up-regulation of NF-κB, among other signaling molecules, we specifically detected up-regulation of Ser473-phosphorylated Akt (AKTpSer473), which suggests AKT signaling might contribute to the function of CICs in WT Xn. (More information on the unique properties of NCAM^+^ cells is presented in Supporting Information Movies S1, S2, Table S4 and Fig S3C).

**Table 3:** Summary of WT formation in NOD-SCID mice by WT Xn derived sorted cells

Tumour code	Cells injected	Tumours/injection									
		
		200	500	1 × 10^3^	1.5 × 10^3^	2.5 × 10^3^	5 × 10^3^	7.5 × 10^3^	1 × 10^4^	4 × 10^4^	>1 × 10^5^
W011	NCAM^+^	–	2/3	–	2/3	–	–	–	3/3	–	–
	NCAM^−^	–	0/3	–	0/3	–	–	–	0/3	–	–
	Non-sorted	–	–	–	–	–	–	–	1/2	–	1/2
W013	NCAM^+^	–	0/3	1/3	–	–	–	–	–	–	–
	NCAM^−^	–	0/3	0/3	–	–	–	–	–	–	–
	Non-sorted	–	0/1	0/1	–	–	–	–	–	–	2/2
W014	NCAM^+^	–	2/4	3/4	–	–	1/1^a^	–	–	–	–
	NCAM^−^	–	0/4	0/4	–	–	0/1^a^	–	–	–	–
	Non-sorted	–	0/2	1/3	–	–	–	–	0/2	2/2^b^	2/2
W013	NCAM^+^PSA-NCAM^+^	–	–	1/2	–	–	2/2	–	3/3	–	–
	NCAM^+^PSA-NCAM^−^	–	–	1/2	–	–	2/2	–	3/3	–	–
	Non-sorted	–	–	–	–	–	1/1	–	–	–	4/4
W011	NCAM^+^CD44^+^	0/2	–	–	–	2/3	–	–	–	1/2	–
	NCAM^+^CD44^−^	0/2	–	–	–	1/3	–	–	–	0/2	–
	Non-sorted	0/2	–	–	–	–	–	–	–	–	2/2^d^
W013	NCAM^+^CD44^+^	–	0/3	–	2/3	–	–	1/3	–	–	–
	NCAM^+^CD44^−^	–	0/3	–	0/3	–	–	0/3	–	–	–
	Non-sorted	–	0/3	–	–	–	–	0/1	–	–	2/2
W014	NCAM^+^CD44^+^	0/2	–	–	–	–	2/2	–	–	1/2^c^	–
	NCAM^+^CD44^−^	0/2	–	–	–	–	0/2	–	–	1/2^c^	–
	Non-sorted	0/2	–	–	–	–	–	–	–	1/2	–
W011	NCAM^+^ALDH1^+^	5/8*	6/11	8/11	–	–	–	–	2/3	–	–
	NCAM^+^ALDH1^−^	0/8	0/11	0/11	–	–	–	–	0/3	–	–
	Non-sorted		0/1	2/5	–	–	–	–	1/3	–	2/2
W013	NCAM^+^ALDH1^+^	1/4*	1/4	3/4	–	–	2/3^a^	–	–	–	–
	NCAM^+^ALDH1^−^	0/4	0/4	0/4	–	–	0/3^a^	–	–	–	–
	Non-sorted		–	2/4	–	–	0/2^a^	–	1/1	–	1/1
W014	NCAM^+^ALDH1^+^	4/6*	–	–	–	–	–	–	–	–	–
	NCAM^+^ALDH1^−^	0/6	–	–	–	–	–	–	–	–	–
	Non-sorted	–	–	1/2	–	–	–	–	1/1	–	1/1

a 4 × 10^3^ cells were injectedl.

b 2.5 × 10^4^ cells were injected.

c 5 × 10^4^ cells were injected.

d 6 × 10^4^ cells were injected.

* *p* = 0.008 for accumulated data.

These results implicated NCAM as a useful marker for prospective isolation of a cell sub-population enriched for WT-cancer initiating activity.

### Aldehyde dehydrogenase 1 (ALDH1) expression defines the CIC/CSC within the NCAM^+^ cell population

**Figure 3:**
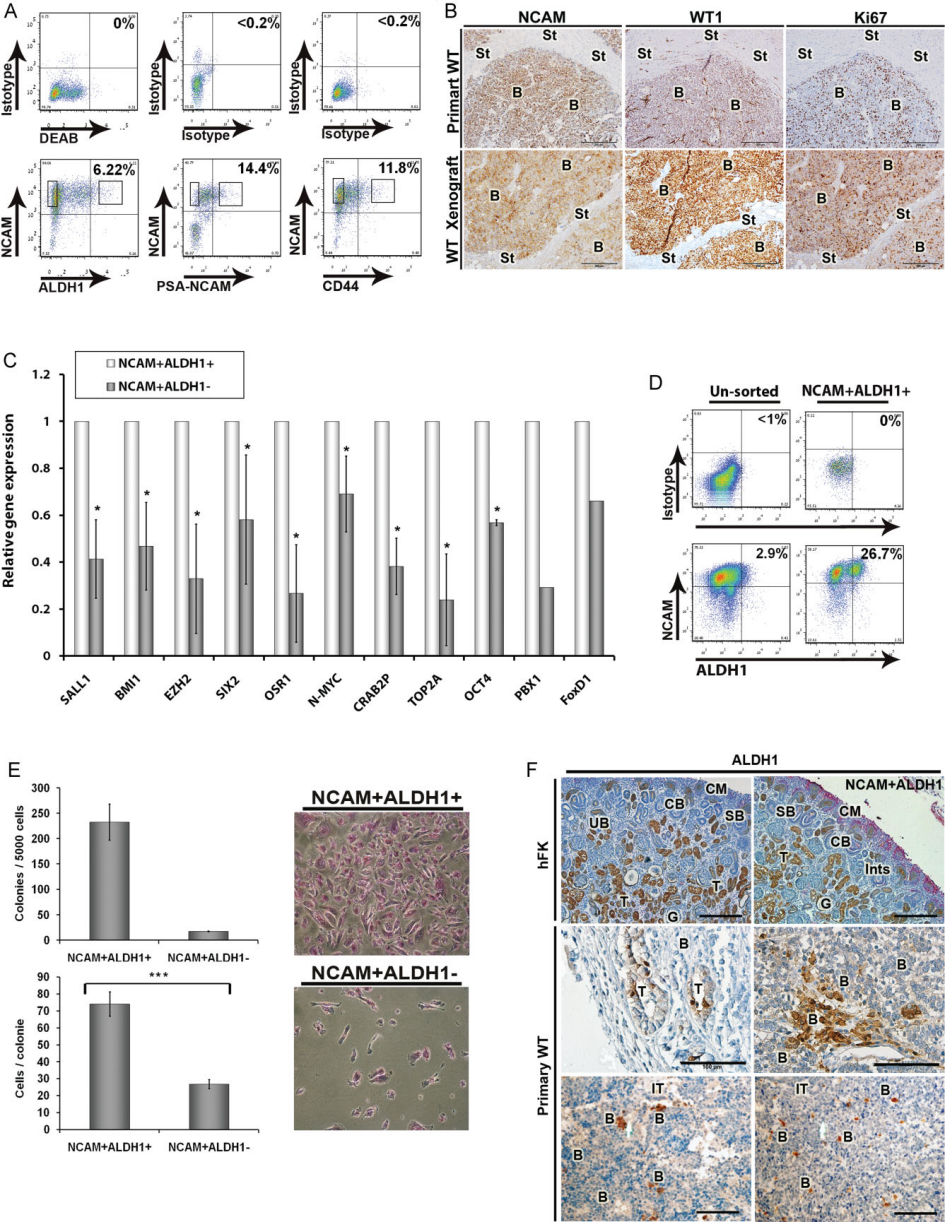
Analysis of additional markers within the NCAM^+^ cell population reveals that ALDH1 activity identifie a CIC/CSC population Representative FACS analysis showing PSA-NCAM, CD44 and ALDH1, to be expressed or active solely in cells within the NCAM^+^ population (See also Supporting Information Fig S4).Immunohistochemical staining presented in serial sections of tumours formed by NCAM^+^ALDH1^+^ cells (lower panels) and of their primary tumour source (upper panels) for NCAM, WT1 and Ki67, shows recapitulation of the parental tri-phasic tumour histology in NCAM^+^ALDH1^+^ derived Xn. (Scales bars = 200 µm; Magnification 20×).qRT-PCR analysis of renal progenitor, stemness and poor prognostic genes in NCAM^+^ALDH1^+^ and NCAM^+^ALDH^−^ WT Xn cells, demonstrates significantly higher mRNA levels of these genes in NCAM^+^ALDH^+^ (*p = 0.031). All values were normalized to NCAM^+^ALDH1^+^ cells (therefore = 1. Results are presented as the mean ± SEM of five separate experiments.Representative FACS plots of ALDH1 activity and NCAM expression in p-WT Xn generated from unsorted cells and Xn generated from the same p-WT Xn (W014) sorted according to NCAM expression and ALDH1 activity (NCAM^+^ALDH1^+^ cells). Plots show an enrichment for NCAM^+^ALDH1^+^ cells in the sorted compared to unsorted xenografts, as well as the appearance of cells lacking ALDH1 activity (ALDH1^−^) that originate from NCAM^+^ALDH1^+^ cells.Colony forming ability was compared between NCAM^+^ALDH1^+^ cells and NCAM^+^ALDH1^−^ WT cells. Number of colonies formed by NCAM^+^ALDH1^+^ cells was higher than that from NCAM^+^ALDH1^−^ cells (top bar graph). Number of cells/colony was significantly higher in NCAM^+^ALDH1^+^ compared to NCAM^+^ALDH1^−^ cells (bottom bar graph; ***p = 0.000017). Representative phase-contrast images of colonies formed from NCAM^+^ALDH1^+^ and NCAM^+^ALDH1^−^ cells are presented on the right. Experiments were repeated twice in triplicates.Immunohistochemical staining for NCAM (red) and ALDH1 (brown) of foetal kidney shows a predominant expression of NCAM in the cap mesenchyme with a distinct expression of ALDH in tubules. In primary WT, ALDH1 is expressed by some immature tubular structures and also by a small number of cells within the blastema. (CM-Cap mesenchyme; SB-S shaped body; CB-Coma shaped body; G-glomerulous; Ints-Interstitium; T-Tubules; B-Blastema; IT-Immature Tubules, Scales bars are indicated in the figure).

Since the NCAM^+^ population is expanded in p-WT Xn, we sought to determine an additional marker to improve the definition of CICs within the NCAM^+^ population. We therefore analysed the expression of additional surface molecules in p-WT Xn cells by FACS. This survey revealed that PSA-NCAM (PSA), the embryonic form of NCAM (10%; Roth et al, 1988a, b) as well as CD44 (10–20%) and aldehyde dehydrogenase (ALDH1) activity (4–10%), that have recently emerged as markers for CIC/CSC in several malignancies (Al-Hajj et al, 2003; Ginestier et al, 2007) were solely contained within the NCAM^+^ cell population (Fig 3A and Supporting Information Fig S4). We therefore initially isolated NCAM^+^PSA-NCAM^+^ or NCAM^+^CD44^+^ cell fractions from p-WT Xn by sorting and analysed their tumour initiating capabilities in mice as well as additional stemness characteristics. We found that these cell fractions did not enrich for WT-initiating activity/CSCs within the NCAM^+^ WT cells (Table 3 and Supporting Information Fig S4). We next sorted p-WT Xn according to NCAM expression and ALDH1 activity to generate NCAM^+^ALDH1^+^ *versus* NCAM^+^ALDH1^−^ cell fractions. In contrast to PSA-NCAM or CD44, NCAM^+^ALDH1^+^ cells reduced the number of p-WT Xn cells required to initiate tumours in mice to as few as 200, while NCAM^+^ALDH1^−^ cells lacked this ability, (Table 3). Histology and immunohistochemistry revealed that these tumours recapitulated the tri-phasic WT phenotype and demonstrated widespread expression of the cell proliferation marker Ki-67 (Fig 3B). qRT-PCR of NCAM^+^ALDH1^+^ cells demonstrated, in addition to their highly enriched WT-initiating activity, significant elevation of early renal progenitor transcripts, stemness factors and WT poor prognostic factors compared to NCAM^+^ALDH1^−^ cells (Fig 3C), indicating that this smaller cell fraction might be responsible for the expression profile observed in the wider NCAM^+^ cell population confounded by other cell phenotypes (NCAM^+^ALDH1^−^). We also observed NCAM^+^ALDH1^+^ cells to overexpress PBX1, a renal stromal lineage marker (Schnabel et al, 2003). FACS analysis of tumours that were immediately initiated by NCAM^+^ALDH1^+^ cells revealed approximately 20% of ALDH1^+^ cells (higher percentage than unsorted p-WT Xn cells which contained 4–10% ALDH^+^ cells) and a large fraction of ALDH1^−^ cells supporting the differentiation capacity of injected ALDH1^+^ cells. (Fig 3D and Supporting Information Fig S5C). Colony forming assays showed significantly higher number of clones and larger colonies in NCAM^+^ALDH1^+^ compared to NCAM^+^ALDH1^−^ cells in accordance with the CSC phenotype (Fig 3E). In addition, xenograft tumours initiated from NCAM^+^ALDH1^+^ cells were further sorted into NCAM^+^ALDH1^+^ and NCAM^+^ALDH1^−^ WT cells and injected to secondary recipients (s.c. in NOD-SCID or NOG mice), in serial dilutions. Consequently, only the NCAM^+^ALDH1^+^ were capable of tumour initiation suggestive of their *in vivo* self-renewal capacity (Table 4). Having elucidated that ALDH1 pinpoints a smaller cell population within the NCAM^+^ fraction capable of initiating and propagating WT, we immunostained human foetal kidneys and primary WT for NCAM1 and ALDH1 to determine possible lineage relationships. In the human foetal kidney, NCAM1, as previously reported, was found to predominantly localized to the capping mesenchyme of the nephrogenic zone (Roth et al, 1988a, b). In contrast, ALDH1 localizes to mature tubules and is absent from the nephrogenic zone in the human foetal kidney (Fig 3F upper panel), while in WT it shares localization in both distinct cells of blastema as well as differentiated tubular elements (Fig 3F, lower panels). Thus, while NCAM is likely to serve as the onco-developmental marker of WT CIC, ALDH1 may be most relevant to the CIC phenotype (Armstrong et al, 2004; Chen et al, 2009; Ginestier et al, 2007; Ma et al, 2008; Metsuyanim et al, 2009; Roth et al, 1988a, b) with no specific connection to nephrogenesis.

**Table 4:** Self renewal – summary of WT formation in NOD-SCID and NOG mice by sorted NCAM+ALDH+ p-WT Xn derived cells

Number of cells injected	Type of cells injected	Tumours/injection			*p*-values
			
		NOD-SCID mice		NOG mice	
				
		W011 p-WT Xn	W013 p-WT Xn	W011 p-WT Xn	
200	NCAM^+^ALDH1^+^	2/4	1/4	3/4	<200 cells
	NCAM^+^ALDH1^−^	0/4	0/4	0/4	0.007
500	NCAM^+^ALDH1^+^	3/4	1/4	2/4	<500 cells
	NCAM^+^ALDH1^−^	0/4	0/4	0/4	0.0002
1 × 10^3^	NCAM^+^ALDH1^+^	3/4	3/4	3/4	<1000 cells
	NCAM^+^ALDH1^−^	0/4	0/4	0/4	
	Non-sorted	2/4	2/4	–	0.0001

### Specific regulatory pathways in NCAM^+^ALDH1^+^ WT Xn CICs provide insight into WT oncogenesis

**Figure 4:**
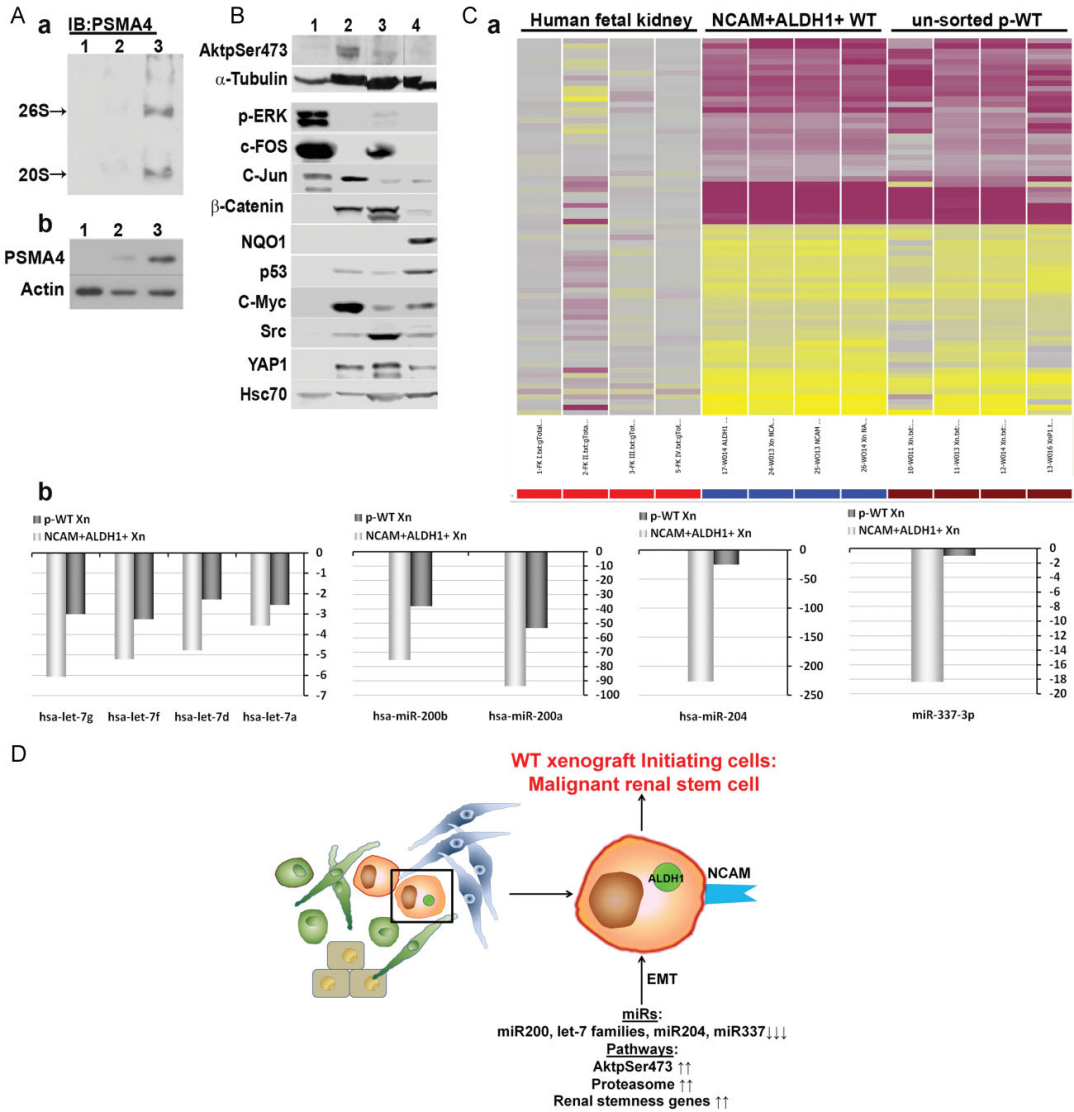
Specific regulatory pathways in NCAM^+^ALDH1^+^ WT CICs provide insight into WT oncogenesis Proteasomal levels were examined in a primary tumour (1) compared to un-sorted p-WT Xn (2) and p-WT Xn generated from NCAM^+^ALDH1^+^ cells (3), showing increased levels in the latter. Proteasomal complexes were analysed by (a) nondenaturing page analysis and total proteasomal subunit protein levels and by (b) immunoblot analysis of polyacrylamide-SDS gel.Immunoblot analysis of polyacrylamide–SDS gel for the expression of the following proteins: AKTpSer473, p-ERK, c-FOS, c-JUN, β-Catenin, NQO1, p53, c-MYC, SRC and YAP1 in parental tumour (1), NCAM^+^ALDH1^+^ derived Xn (2), foetal kidney (3) and rhabdoid tumour (4). Showing that AKTpSer473, but not p-ERK and c-FOS, are differentially expressed in NCAM^+^ALDH1^+^ derived p-WT Xn.Agilent microRNA microarray comparison of un-sorted p-WT Xn, NCAM^+^ALDH1^+^ derived p-WT Xn and human foetal kidneys. (a) Heat map of 71 miRNAs differentially expressed between p-WT Xns and human foetal kidneys. Expression pattern was similar among all p-WT Xns, with 35 down-regulated and 36 up-regulated microRNAs (miRs) compared to the hFK tissues. (b) Expression of microRNA families let-7 and 200 (miRs 200a and 200b), miR-204 and miR-337 is markedly reduced in NCAM^+^ALDH1^+^ derived p-WT Xn compared to un-sorted p-WT Xn and human foetal kidney.Suggested schematic representation of the regulatory pathways governing WT CIC phenotype and function. For array experiments four sources (*n* = 4) for each tissue type were used.

We next determined that biological properties of the NCAM^+^ALDH1^+^ CIC/CSCs in WT. Since Xn tumours initiated from NCAM^+^ cells differentially expressed specific proteins such as AKTpSer473 (Fig 2E and Supporting Information Table S4), the protein profile of tumour Xn early after initiation by NCAM^+^ALDH1^+^ cells might provide insights into CIC/CSCs biology. Nondenaturing PAGE and immunoblot analysis for the expression of proteasomal subunits 19s and 20s revealed overexpression of the subunits in comparison to both tumour xenografts generated from unsorted p-WT Xn cells and parental WT (Fig 4A). Moreover, Western blot analysis demonstrated differential protein expression in the NCAM^+^ALDH1^+^ tumour, specifically AKTpSer473 but not p-ERK and c-FOS, which have been recently implicated in WT and were strongly expressed in the primary parental WT (Fig 4B). c-Jun seemed to be activated in WT in general while β-catenin was similarly observed in NCAM^+^ALDH1^+^ tumour and hFK and therefore does not necessarily specify CIC pathways. c-MYC, SRC and YAP1 were expressed in most samples (WT and control rhabdoid tumours) with c-MYC elevated in NCAM^+^ALDH1^+^ tumour. Other proteins, such as NQO1, and to a lesser extent P53, were increased in control paediatric rhabdoid tumour, further indicating the specificity of the pathways active in WT and importantly in the NCAM^+^ALDH1^+^ CIC derived tumour xenografts. Thus, highly enriched WT CIC/CSCs are likely to feature signaling molecules and oncogenes distinct from those generally observed in WT and other closely related paediatric tumours.

To gain further insight into regulatory processes in WT CICs, human Agilent miRNA arrays covering 1349 small noncoding RNAs were applied to tissue extracts of unsorted p-WT Xn, NCAM^+^ALDH1^+^ derived p-WT Xn and human foetal kidney, resulting in the identification of 71 statistically significant, differentially expressed microRNAs (Supporting Information Table S5 and Fig S5D). The expression pattern was similar among all WT Xns, with 35 down-regulated and 36 up-regulated microRNAs (miRs) compared to the hFK tissues (Fig 4C and Supporting Information Table S5). Importantly, the most significantly reduced microRNAs families: let7 and 200, as well as miR-204, are related to control of epithelial cell differentiation and epithelial–mesenchymal transition (EMT; Gregory et al, 2008; Li et al, 2009; Paterson et al, 2008; Peter, 2009; Wang et al, 2010) while miR-337 has been linked to apoptosis, (Ovcharenko et al, 2007) osteoblast differentiation and was shown to be epigenetically silenced in oral cancers (Kozaki et al, 2008). Moreover, further analysis revealed that changes in miR expression were enhanced in NCAM^+^ALDH1^+^ derived p-WT Xn compared to unsorted p-WT Xns. Accordingly, microRNA family let-7, miR-200a and miR-200b, miR-204 and miR-337 showed approximately 2-, 8- and 18-fold reduction, respectively, in NCAM^+^ALDH1^+^ derived p-WT Xn compared to unsorted p-WT Xn and a larger reduction when compared to human foetal kidneys (Fig 4Cb, Supporting Information Fig S5D and Table S5). Thus, tumour extracts enriched with NCAM^+^ALDH1^+^ cells revealed a microRNA signature that indicates the highest degree of loss of epithelial differentiation and EMT, providing new insight into the pathophysiology of WT (Fig 4D).

### First line chemotherapy fails to eradicate WT CIC/CSCs *in vitro*

WT is currently treated both pre- and post-operatively with a combination of the two chemotherapic drugs vincristine and actinomycin D, to which other drugs may be added (Green et al, 1996). Relapsing WT patients have reduced survival rates and often show resistance to conventional first line treatment (Pinkerton et al, 1991). A combination of the topoisomerase II inhibitor, etoposide and cisplatin improves survival rates in these patients (Sparano et al, 1991). Therefore, following calibration of IC50 for chemotherapeutic combination and treatment of primary WT cells in culture, we analysed by FACS the changes in NCAM^+^ALDH1^+^ cells in comparison to untreated controls. We found a substantial reduction in the NCAM^+^ALDH1^+^ fraction only after second-line WT chemotherapy treatment (Supporting Information Fig S6).

### *In vivo* targeting of NCAM^+^ WT cells with a humanized anti-NCAM antibody-drug conjugate (lorvotuzumab-mertansine) eliminates WT xenografts

**Figure 5:**
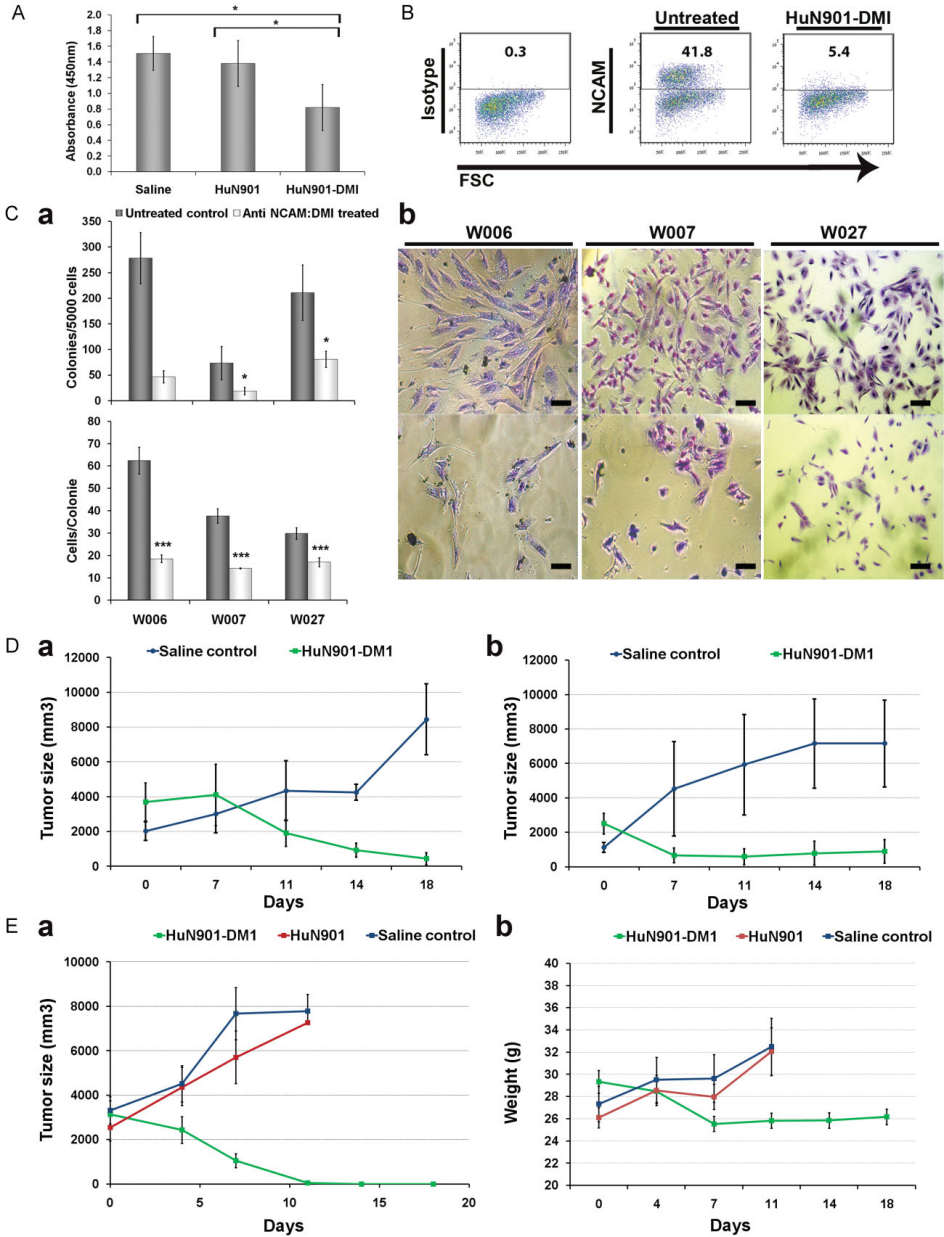
Specific targeting of NCAM^+^ WT cells with lorvotuzumab-mertansine induces *in vivo* eradication of WT xenografts Anti NCAM specific effect of lorvotuzumab-mertansine on WT cell survival and stemness properties. Cells derived from three different WT Xn (W016, W027 and W028) were exposed to the immunoconjugate, unconjugated huN901 antibody or saline. Cell survival was significantly reduced in the lorvotuzumab-mertansine treated compared with HuN901 or saline treated controls. Cell survival was measured using an MTS assay. Data is presented as mean ± SEM of triplicate experiments from the three different WT Xn sources. *p = 0.03 from saline and *p* = 0.034 from unconjugated HuN901 controls.FACS analysis for NCAM expression in cultured W011 WT Xn-derived cells either treated with 0.18 µM lorvotuzumab-mertansine or with saline for 5 days. NCAM expressing cells were preferentially eliminated from the W011 cell population exposed to lorvotuzumab mertansin.Colony forming ability (CFU) was compared between primary WT cells from three different patients (W006, W007, W027) either treated with 0.18µM lorvotuzumab-mertansine or untreated control for 5 days. (a – upper panel) Number of colonies formed by lorvotuzumab-mertansine treated cells was significantly lower than that of untreated cells (*p = 0.08, 0.02, 0.04, respectively). (a – lower panel) The number of cells per colony was significantly lower in Ab treated compared to untreated cells. Data is presented as mean ± SEM of at least two experiments performed in triplicates. (*p = 3E−06, 4E−06, 0.0002, respectively). (b) Representative phase-contrast images of colonies formed from treated and untreated WT cells from three patients (Scale bars = 100 µm). *In vivo* activity of huN901-DM1 in tumour xenograft models of human WT.Mice were treated intravenously with either (1) normal saline; (2) lorvotuzumab-mertansine (360 µg DM1/kg or 18 mg conjugate/kg). Tumour volume was assessed in three dimensions using an electronic caliper once-twice a week (using the formula *V* = *L* × *W* × *H*), and the volume is presented in mm^3^. (a) Mice bearing palpable xenografts (W011 Xn, W013 Xn and W014 Xn) that contained high NCAM levels (HNWT) and were treated with saline (*n* = 6), showed exponential tumour growth, while those treated with lorvotuzumab-mertansine (*n* = 10) demonstrated complete or near complete tumour eradication 3 weeks after the initial treatment was administered (*p* = 0.03). (b) Mice bearing LNWT treated with saline (*n* = 3) showed similar results, while treatment with lorvotuzumab-mertansine (*n* = 4), showed significant initial reduction in tumour volume (>50%) followed by a plateau (*p* = 0.03).(a) Mice bearing palpable tumours were randomized into three treatment groups: (1) Lorvotuzumab-mertansine (360 µg DM1/kg; *n* = 15); (2) unconjugated Ab (HuN901-13.3 µg/kg; *n* = 12); (3) saline as a control (*n* = 11). Tumours treated with Lorvotuzumab-mertansine were completely eradicated following 3 weeks of treatment while tumours treated with either unconjugated Ab or saline showed rapid tumour growth (*p*-values are 1.29E−07 and 5.75E−08, respectively). (b) Mouse weights during treatment.

Based on our observation that the NCAM^+^ cell fraction was enriched for WT-initiating activity and contained WT Xn CICs, as well as on the fact that chemotherapy might not efficiently target the WT CIC population, (Campbell et al, 2004; Pode-Shakked et al, 2009), we performed proof-of-principle experiments to evaluate the therapeutic effects of lorvotuzumab-mertansine, an anti-NCAM antibody-cytotoxic drug conjugate currently in clinical development (Jensen & Berthold, 2007; Ricart, 2011). First, we determined the *in vitro* IC50 for this compound on WT Xn (W011) populations in which approximately 80% of cells express NCAM. As an antigen-negative control, we used the DLD cell line, which does not express NCAM (Supporting Information Fig S7). All consequent *in vitro* assays were performed using fourfold the IC50 to ensured eradication of all NCAM^+^ cells in the culture. WT cell death was significantly higher following application of lorvotuzumab-mertansine in comparison to treatment with the unconjugated antibody (HuN901) or with saline only (Fig 5A). To examine whether lorvotuzumab-mertansine targeted preferentially NCAM-expressing cells within the W011 Xn cell populations, we compared NCAM expression in untreated and treated W011 cell populations by FACS analysis. These experiments were conducted using the allophycocyanin (APC)-labeled anti-NCAM (anti-CD56) antibody MEM188 that binds to an epitope that is not blocked by HuN901 antibody binding. We observed a dramatic reduction in NCAM expression after treatment of W011 cells with lorvotuzumab-mertansine from 42 to 5% (Fig 5B). In accordance, there was a significant reduction in colony forming capacity of the WT cell cultures following treatment (Fig 5Ca) and fewer cells per colony (Fig 5Ca lower panel and 5Cb). These results suggested that lorvotuzumab-mertansine specifically targets the NCAM^+^ WT cell fraction. We next examined the anti-tumour activity of lorvotuzumab-mertansine on WT subcutaneous xenograft models in NOD/SCID mice. We utilized four xenograft sources established in 60 mice, three of which (W011 Xn, W013 Xn and W014 Xn) with high NCAM expression levels (HNWT), with an average of 90, 72 and 84% positive cells, respectively, and one (W016 Xn) with a relatively low NCAM expression level (LNWT) with an average of 50% NCAM-positive cells. For the initial experiments mice bearing tumours of each tumour type were divided into three treatment groups: (1) high doses of lorvotuzumab-mertansine (360 µg DM1/kg or 18 mg conjugate/kg); (2) low doses of lorvotuzumab-mertansine (180 µg DM1/kg or 9 mg conjugate/kg); (3) saline control. All groups were treated intravenously once a week for three consecutive weeks and monitored for tumour growth once-twice a week. Preliminary results determined the optimal dose (360 µg DM1/kg or 18 mg conjugate/kg), which was used in all subsequent larger scale experiments. To determine the effect of lorvotuzumab-mertansine on both HNWT and LNWT, mice were randomized into saline treated and lorvotuzumab-mertansine treated groups (Fig 5D). In the control mice (*n* = 9) injected with saline, both HNWT and LNWT tumours grew in an exponential pattern. A complete to nearly complete regression of tumours was observed in mice bearing HNWT (*n* = 10) after 3 weeks of lorvotuzumab-mertansine injections (Fig 5Da). In treated mice bearing LNWT (*n* = 4), we detected an initial significant reduction of tumour size (>50%) followed by a plateau, possibly due to residual NCAM^−^ cells that remained post treatment but lacked WT-initiating activity and hence failed to resume tumour growth (Fig 5Db). We next randomized mice bearing palpable tumours into three treatment groups: (1) lorvotuzumab-mertansine (360 µg DM1/kg; *n* = 15); (2) unconjugated antibody (HuN901-13.3 µg/kg; *n* = 12); (3) saline as a control (*n* = 11). Tumour treated with lorvotuzumab-mertansine were completely eradicated following 3 weeks of treatment while tumours treated with HuN901 or saline showed rapid tumour growth (Fig 5Ea). Comparison of mice weights revealed that while weight increased dramatically in both saline and unconjugated Ab treatment groups as a result of rapid tumour growth, immunoconjugate treated mice underwent weight loss during the first 2 weeks following total eradication of the tumours. However, in the 3rd week weights stabilized, indicating the lack of toxic effects caused by the treatment (Fig 5Eb). Intriguingly, of all mice treated with lorvotuzumab-mertansine two tumours relapsed after response. FACS analysis of NCAM and ALDH1 levels in these tumours revealed the presence of a NCAM^+^ALDH1^+^ cell population, possibly explaining their re-appearance (Supporting Information Fig S8). Treatment of these relapsed tumours with lorvotuzumab-mertansine at the initial dose (360 µg DM1/kg or 18 mg conjugate/kg) resulted in their complete eradication.

## DISCUSSION

Using a WT xenograft model, we have identified a cell sub-population that unequivocally functions as human tumour-initiating cells/cancer stem cells. We generated WT xenografts that are continually initiated and propagated in mice by serial cell transplantation and recapitulated the primary tumour's tri-lineage morphology. While being able to form all lineages in the tumour (blastema, stroma and epithelium), close examination of the propagatable xenografts revealed a shift towards highly proliferating blastemal predominant tumours that overexpressed renal stemness genes and those known to predict poor outcome in WT patients. The validity of using these xenografts is supported by clinical observations that tumours with a predominant blastemal component are aggressive, while tumours with blastemal predominance after chemotherapy are classified as “high risk” and patients with blastemal predominant tumours after chemotherapy have a high relapse and low survival rates (Kaste et al, 2008; Reinhard et al, 2008). Thus, the establishment of human WT in immunodeficient mice selected and expanded a more aggressively malignant phenotype, reminiscent of the process of aggressive disease and relapse in patients.

While the serial propagation of human WT by unsorted cells may serve by itself as a method to enhance a more tumourigenic CIC/CSC phenotype, we failed, even in later generation xenografts, to go below 10,000 cells for continual WT initiation. After prospective isolation of the NCAM^+^ fraction, however, tumour initiation could be obtained from as few as 500 cells. Our own previously published immuno-histochemical analysis of primary human WT sections, localized NCAM predominantly to the blastemal component indicating that an undifferentiated “blastemal cell” and NCAM^+^ cell might be interchangeable (Pode-Shakked et al, 2009; Roth et al, 1988a, b). Nevertheless, a “blastemal” cell has never before been shown to generate a tri-phasic WT and qualify for the functional definition of CIC/CSC. Thus, although NCAM was relatively abundant in the blastema predominant p-WT Xn (it is significantly less frequent in primary tri-phasic WTs), functional experiments showed the NCAM^+^ population to unequivocally contain enhanced WT-cancer initiating activity, while NCAM^−^ cells did not. Indeed, divergent and high frequencies of tumourigenic cells with enriched cancer-initiating activity have been suggested for certain malignancies (Quintana et al, 2008).

While blastemal cells appear as a homogeneous population, we further analysed cell fractions within the NCAM^+^ population and discovered that 200 purified NCAM^+^ALDH1^+^ cells were able to initiate WT xenografts, recapitulate the heterogeneous phenotype of their parental WT and further propagate in mice, reflecting both the differentiation and self-renewal capacities of CSCs (Li et al, 2007). ALDH1 has recently been shown to play a pivotal role in mediating chemo resistance in CSCs where it acts to detoxify harmful substances such as aldehydes that would otherwise damage stem/progenitor cells (Dylla et al, 2008; Tanei et al, 2009). High ALDH1 activity levels have been used to better identify CSCs in a variety of tumours including liver (Ma et al, 2008), head and neck (Chen et al, 2009), colorectal (Dylla et al, 2008) and breast (Ginestier et al, 2007) tumour-initiating populations. The localization of ALDH1 to a fraction of WT blastema cells but not to the undifferentiated blastema of the human foetal kidney (located in differentiated structures), indicates that ALDH1 is likely to be associated with stem cell activity and CSCs, similarly to other cancers (Ginestier et al, 2007; Ma et al, 2008; Pearce et al, 2005; Silva et al, 2011), rather than being a specific renal lineage blastema marker (Metsuyanim et al, 2009; Roth et al, 1988a, b).

The discovery of CIC/CSCs in WT Xns provided the proof-of-principle that differences in signaling pathways between CICs and the general tumour cell population do exist. Analysis of tumour xenografts generated from the sorted CIC/CSC population early after initiation, with a protein array/Western blotting disclosed few preferentially-expressed pathway components with protein kinase B (PKB)/Akt signaling signified in all of our analyses. Of note, it is now apparent that the Akt/mTOR signaling network plays a key role in cancer stem cell biology and that CIC/CSCs display preferential sensitivity to the inhibition of this pathway (Martelli et al, 2010), a feature that can be exploited in future investigations of WT CIC/CSCs. Moreover, global microRNA analysis showed specific microRNAs to be differentially expressed identifying a strong miRNA signature for the NCAM^+^ALDH1^+^ WT CICs. We found that microRNA-200 family, let-7 family and miR-204 were markedly downregulated in propogated WT xenografts but to a greater extent in those Xn initiated by, and enriched for, NCAM^+^ALDH^+^ cells (miR-204 was reduced 226-fold compared to hFK). These microRNAs have been closely linked with epithelial differentiation or maintenance and regulation of EMT in several malignancies (Gregory et al, 2008; Kong et al, 2011; Li et al, 2009). EMT has been shown to transform early tumours into invasive, metastatic and recurrent neoplasms and to be closely associated with a cancer stem cell phenotype (Mani et al, 2008). In fact, in inducible oncogenesis models, the miR-200 family is inhibited during CIC/CSC formation (Iliopoulos et al, 2010). Furthermore, the PKB/Akt pathway activated in WT CICs has been shown to regulate the EMT process independently (Grille et al, 2003) or by modulation of the miR-200 family (Iliopoulos et al, 2009). Taken together, these observations suggest that EMT and lack of proper mesenchymal–epithelial transition are likely to promote WT CIC/CSC function, generating highly tumourigenic undifferentiated cancer cells with a mesenchymal phenotype and contribute to WT propagation *in vivo*. Expression of mesenchymal cell regulators that prevent epithelial differentiation and enhance self-renewal of renal stem/progenitor cells (*OSR1, SIX2*) alongside the acquisition of stemness through expression of pluripotent (*OCT4*) and Polycomb (*BMI1, EZH2*) genes (Kashyap et al, 2009), all found to be overexpressed in WT Xn CICs, may contribute to this phenotype.

Our findings with respect to the CIC population may provide insight into the cell lineage of origin for WT. Given our observation that WT Xn CICs can give rise to blastemal, stromal and epithelial elements (including tubular and glomeruloid bodies, indicative of abortive nephrogenesis), one can assume a common undifferentiated renal mesenchymal cell of origin for all three cell types. Indeed, it is generally accepted that both the stroma and the nephron progenitors arise from the metanephric mesenchyme (Kobayashi et al, 2008; Oliver et al, 2002). In lineage studies, Osr1^+^ intermediate mesoderm is shown to give rise to almost all cell populations within the kidney, including stromal/interstitial cells as well as the nephron progenitor population, the cap mesenchyme (Mugford et al, 2008). We found that WT Xn CICs were enriched for nephron progenitor markers (*SIX2/SALL1/OSR1*) and that there was an intimate link between WT-initiating activity and enrichment level for these genes. In addition, a number of the genes found to be elevated in the WT CIC population (*SALL1, NCAM*) are expressed in the Osr1^+^ metanephric mesenchyme and are not restricted to the nephron progenitor population (Evseenko et al, 2010; James et al, 2006; Metsuyanim et al, 2009). Similarly, PBX1, enriched in WT CICs, is expressed in the metanephric mesenchyme that gives rise to both stroma and epithelium. This would support a simplified model in which the WT CIC population represents a transformed intermediate mesoderm/metanephric mesenchymal cell prior to EMT.

Alternatively, WT CICs may relate to the more committed nephron progenitor cells, a population shown to self-renew and differentiate exclusively to nephron epithelia throughout nephrogenesis, fulfilling the criteria of a true committed stem cell (Kobayashi et al, 2008). In this scenario, the nephron stem/progenitor cell would give rise to partial nephrogenesis (tubular and glomeruloid bodies) but also assume a stromal and primitive mesenchymal fate through the trans-differentiation/de-differentiation signals illustrated above. Finally, the WT CIC may represent an entity entirely distinct from the tumour cell of origin. In this regard, transformation studies of human renal stem/progenitor populations and differentiated cell types derived from the developing kidney leading to WT initiation and oncogenesis or transgenic WT animal models (Hu et al, 2011) are likely to clarify the relationship between the two.

Perhaps the most important translational contribution of human CSC/CIC research is that it unveils specific biomarkers that can be targeted *in vivo* by antibody therapy to disrupt tumour growth. The most investigated example is the acute myeloid leukaemia (AML) CSCs, isolated more than a decade ago (Bonnet & Dick, 1997) leading to further identification of markers for targeting hematopoietic cancer stem/progenitor cells (Chan et al, 2009; Jin et al, 2006). Many biomarkers have been known to be expressed in specific cancers long before their implication in CIC/CSC activity. Indeed, only later was the eradication of the cancer cell population they identify, proposed as a means to treat human malignancies (Jin et al, 2006; Naor et al, 1997). Accordingly, NCAM which was known to be expressed in WT is also a marker for functional WT-initiating activity; hence the importance of its targeting. This is also supported by our data showing that 1st line chemotherapy used to treat WT patients is less effective on NCAM^+^ or NCAM^+^ALDH1^+^ cells *in vitro* while 2nd line drugs used to treat WT relapse have a more profound effect but do no eradicate all WT CICs. This data should be interpreted in light of the fact that the IC50 of these drugs limits escalation of drug dosage *in vitro*, while *in vivo* treatment might allow a higher dosage bearing an effect on the CIC population even by first-line chemotherapies. Indeed, current chemotherapy regimens in WT patients, which cure 85% of WT patients, are employed at doses resulting in, among other adverse effects, devastating secondary malignancies occurring in adulthood (Geenen et al, 2007; Moss et al, 1989; Vane et al, 1984). Therefore, from a clinical standpoint, a combined regimen involving the eradication of the WT-initiating activity via targeting the NCAM molecule might prove useful in reducing chemotherapy toxicity in these patients, as well as in those that do not respond to conventional treatment or have a relapsing disease. Our proof-of-principle experiments targeting this human cell fraction with the anti NCAM antibody-drug conjugate lorvotuzumab-mertansine, might ultimately pave the way for such an approach. In our experiments, lorvotuzumab-mertansine inhibited the *in vitro* stemness properties (CFU capacity, proliferation) of WT cell cultures that varied in the extent of NCAM expression and the *in vivo* targeting of multiple WT xenograft models lead to dramatic results: WT bearing high NCAM expression were completely or significantly eradicated (*n* = 10). Two tumours that relapsed were associated with elevated NCAM levels and a residual NCAM^+^ALDH^+^ fraction and were completely eradicated by an additional course of lorvotuzumab-mertansine suggesting the NCAM^+^ cells were indeed responsible for tumour recurrence. Treatment of low NCAM expressing WT xenografts (LNWT) with low dose lorvotuzumab-mertansine resulted in attenuation of tumour growth, however without reduction in tumour size, possibly due to incomplete abolition of NCAM^+^ cells harbouring WT-initiating capacity. However, treatment of LNWT with high dose lorvotuzumab-mertansine resulted in reduction of tumour size followed by a plateau suggesting that once all NCAM^+^ cells were eliminated, the remaining NCAM^−^ cells comprising most of these xenografts lacked CIC capacity. Since LNWT xenografts and primary WT mostly harbour similar NCAM levels, utilization of the immunoconjugate to eliminate WT-initiating activity and conventional chemotherapy for non-CIC cancer cells is likely to be more clinically relevant.

We propose that our findings may be relevant to other paediatric solid tumours representing a distinct biological entity, with great similarities among themselves and marked differences from adult counterparts. Accordingly, taking into account that NCAM marks stem/progenitor cell compartments in various developing human organs (Schmelzer et al, 2006; Thomas et al, 2008; Tsokos et al, 1987) and that the corresponding paediatric solid tumours (*e.g.* hepatoblastoma, neuroblastoma and mesodermal tumours as rhabdomyosarcoma) recapitulate development, NCAM targeting might prove useful in a wide spectrum of developmental malignancies.

## MATERIALS AND METHODS

### *In vivo* xenograft experiments

Primary WT samples were obtained from 18 WT patients (Supporting Information Table S1) within 1 h of surgery from both Sheba Medical Center and Hadassah-Ein Kerem Hospital. The local ethical committee approved all studies and informed consent was given by the legal guardians of the patients involved according to the declaration of Helsinki.

Initial WT xenografting to 5–8 weeks old, female, nonobese nondiabetic immunodeficient mice (NOD/SCID) was performed as previously described (Dekel et al, 2006). Briefly, primary WT tissue was cut into 2–5 mm pieces and implanted subcutaneously in the back of the mice. In all the surgical procedures, mice were anaesthetized with i.p. injections of 100 mg/kg ketamine and 10 mg/kg xylazine. Tumours were harvested approximately 3–6 months post implantation or when they reached a size of 1.5 cm diameter, and tumour tissue was processed for immunohistochemical (IHC) staining and formation of single cell suspension. Single cells suspensions were obtained by mincing the samples in Iscove's modification of Dulbecco's medium (IMDM) containing antibiotics (penicillin and streptomycin), followed by treatment with collagenase IV for 2 h at 37°C. Enzymatically treated tissue was triturated using IMDM at twice the volume of the collagenase solution and the suspension filtered (100 µm cell strainer) and washed twice with IMDM containing antibiotics. Erythrocytes were removed by ACK RBS lysis buffer. WT xenograft derived cells thus generated were used for all subsequent assays. Serial transplantation of dissociated cells from freshly retrieved WT Xn tumours was performed by injecting approximately 10^6^ cells in 100 µl 1:1 serum free medium/Matrigel (BD Biosciences, San Jose, CA) subcutaneously into the flanks of 5–8-week old NOD/SCID mice. This procedure was attempted for tumour cells dissociated from the primary tumour as well; however, engraftment rates were less than 10%, in contrast to the 100% engraftment obtained with dissociated cells from WT propagated in Xn. In an attempt to optimize conditions for WT, Xn formation in immunodeficient mice from dissociated single cells, in addition to transplanting freshly dissociated cells, Xn-derived cells were cultured as adherent cultures in Iscove's Modified Dulbecco's Media (IMDM) supplemented with 10% foetal bovine serum (FBS) or as in suspension as “spheres” in serum-free medium (SFM; see below). Cells that had been grown as adherent cultures and those grown as spheres were resuspended in 100 µl SFM/Matrigel and injected into either NOD/SCID or SCID/BEIGE mice in triplicate, as described above. To assess tumourigenicity of WT Xn cell subpopulations dissociated tumour cells were counted, sorted according to the chosen markers, then re-suspended in 100 µl 1:1 SFM/Matrigel, and injected s.c. in serial dilutions into the right (^+^ cells) and left (^−^ cells) flanks of 5–8 week old female NOD/SCID mice. Engrafted mice were inspected bi-weekly for tumour appearance by visual observation and palpation. Mice were sacrificed by CO_2_ inhalation at a tumour diameter of 1.5 cm or at 6-month post transplantation. Following resection, WT xenograft tumours were divided into five pieces, one was fixed and paraffin embedded for histologic staining of NCAM, WT1 and Ki67 to determine recapitulation of the parental tumour phenotype, three small pieces were kept in liquid nitrogen for RNA, DNA and protein extraction and the fifth piece was taken for formation of single cell suspension, followed by serial injections into the flanks of secondary mice (*n* = 3 in each passage). This was repeated at least three times. All animal experiments were conducted in accordance with the National Institutes of Health guidelines for the care and use of animals and with an approved animal protocol from the Sheba Medical Center Animal Care and Use Committee.

### Primary WT and WT Xn cell cultures

Single cell suspensions from primary WT or WT Xn tissues were grown in two culture conditions as mentioned above: in SFM under low attachment conditions where they formed free-floating spheres, or in SCM as adherent cultures for *in vivo* and *in vitro* assays. For sphere formation, single cells were plated in ultra low attachment six-well plates (Corning Life Sciences, Wilkes Barre, PA, USA) at 20,000 cells/well in serum-free DMEM-F12 (Invitrogen), supplemented with 10 ng/ml basic fibroblast growth factor (bFGF) and 20 ng/ml epidermal growth factor (EGF; R&D systems). For adherent growth, cells were cultured on conventional tissue culture plastic in IMDM medium supplemented with 10% FBS and the following growth factors: 50 ng/ml bFGF, 50 ng/ml EGF and 5 ng/ml.

**The paper explained**

PROBLEM:

Cancer initiating cells/cancer stem cells (CICs/CSCs) are thought to be primarily responsible for tumour initiation, their persistence after conventional chemo/radiotherapy may lead to disease recurrence and hence their therapeutic targeting may be essential to ensure complete tumour eradication. WT, the most frequent tumour of the genitourinary tract in children, has served for decades as a general biologic model to study the intersection of cancer and development. Given the considerable differences in tumour biology between adult and paediatric cancers, the existence of cancer initiating and propagating cells in paediatric solid tumours including in WT is questionable. Identification of CICs in human WT is especially challenging due to minimal *in vivo* tumourigenicity of primary WT cells and lack of cell lines, hampering the ability to functionally define CICs via their enriched capacity to regenerate cancers in xenograft mouse models.

RESULTS:

Our article reports the establishment of a human WT xenograft model system mimicking aggressive malignancy in patients and affording the opportunity to isolate undifferentiated “blastemal” CICs in WT, their prospective isolation in line with the functional criteria of CIC/CSCs and in depth molecular characterization showing elevated transcript levels of renal “stemness” and poor WT prognosis genes, preferential protein expression of phosphorylated PKB/Akt and strong reduction of the miR-200 family a known regulator of EMTs. Finally, targeting of a cancer cell population enriched for CIC activity leads to dramatic tumour eradication or attenuation in human WT xenograft models.

IMPACT:

In this paper, we report for the first time the discovery of CIC/CSCs in human WT xenografts, their characterization and therapeutic targeting. This both provides molecular insights into tumour biology and opens practical new venues to explore in the treatment of WT patients; Even though there have been successes in good prognosis WT treatment, our ultimate goal is to reduce the burden of conventional chemotherapy so as to decrease its long-term complications in this patient group. For the group of patients with relapsing bad prognosis disease treatments developed to eradicate CICs may have direct impact on morbidity and mortality.

### Treatment of WT xenograft models with lorvotuzumab-mertansine

WT xenografts were formed in 5–8 week old, female NOD-SCID mice (NOD.CB17-*Prkdc*^*scid*^/NcrCrl**)** by s.c. inoculation of Xn-derived freshly dissociated cells with either high (HNWT - W011, W013, W014 WT Xn) or low (LNWT – W016 WT Xn) NCAM expression. Four weeks later, mice bearing palpable tumours of each tumour type were randomized in the first series of experiments into three groups: saline treated, high dose lorvotuzumab-mertansine treated (360 µg DM1/kg or 18 mg conjugate/kg) and low dose lorvotuzumab-mertansine treated (180 µg DM1/kg or 9 mg conjugate/kg). Each mouse received bolus injections into the tail vein once a week for three consecutive weeks. Tumour size was calculated in three dimensions: *L* (length) × *W* (width) × *H* (height), using a caliper. Tumour dimensions were measured twice a week from the first conjugate injection until either tumours size reached 7000 mm^3^ or mouse death. Following determination of the preferred dose of lorvotuzumab-mertansine (360 µg DM1/kg or 18 mg conjugate/kg), in all the following larger scale experiments, mice were treated and followed as described above. To determine the effect of lorvotuzumab-mertansine on HNWT and LNWT, mice bearing tumours were randomized into two groups, either treated with lorvotuzumab-mertansine or with saline as control. To compare the effect of lorvotuzumab-mertansine with that of the unconjugated form of this Ab (HuN901) as well as to determine possible toxic effects, mice bearing palpable tumours were randomized into three treatment groups: saline, lorvotuzumab-mertansine (360 µg DM1/kg), and unconjugated Ab (HuN901-13.3 µg/kg). During the experiment, mice weights were measured and documented.

### Statistical analysis

Error bars represent the mean ± SEM, unless otherwise indicated. Statistical differences in gene expression (qRT-PCR) between WT cell populations were evaluated using the non-parametric, one-sided Sign test. Statistical differences of all the “*in vivo*” experiments were determined using chi-square test. Statistical differences between additional data groups were determined using Student's *t*-test. For all statistical analysis, the level of significance was set as *p* < 0.05 unless otherwise indicated.

## For more information

http://omim.org/entry/100640

http://omim.org/entry/116930

http://theoncologist.alphamedpress.org/content/10/10/815.long

http://eng.sheba.co.il/PediatricStemCell/

## Supplementary material

Detailed facts of importance to specialist readers are published as ”Supporting Information”. Such documents are peer-reviewed, but not copy-edited or typeset. They are made available as submitted by the authors.

## References

[b1] Al-Hajj M, Wicha MS, Benito-Hernandez A, Morrison SJ, Clarke MF (2003). Prospective identification of tumorigenic breast cancer cells. Proc Natl Acad Sci USA.

[b2] Armstrong L, Stojkovic M, Dimmick I, Ahmad S, Stojkovic P, Hole N, Lako M (2004). Phenotypic characterization of murine primitive hematopoietic progenitor cells isolated on basis of aldehyde dehydrogenase activity. Stem Cells.

[b3] Bonnet D, Dick JE (1997). Human acute myeloid leukemia is organized as a hierarchy that originates from a primitive hematopoietic cell. Nat Med.

[b4] Campbell AD, Cohn SL, Reynolds M, Seshadri R, Morgan E, Geissler G, Rademaker A, Marymount M, Kalapurakal J, Haut PR (2004). Treatment of relapsed Wilms' tumor with high-dose therapy and autologous hematopoietic stem-cell rescue: the experience at Children's Memorial Hospital. J Clin Oncol.

[b5] Chan KS, Espinosa I, Chao M, Wong D, Ailles L, Diehn M, Gill H, Presti J, Chang HY, van de Rijn M (2009). Identification, molecular characterization, clinical prognosis, and therapeutic targeting of human bladder tumor-initiating cells. Proc Natl Acad Sci USA.

[b6] Chen YC, Chen YW, Hsu HS, Tseng LM, Huang PI, Lu KH, Chen DT, Tai LK, Yung MC, Chang SC (2009). Aldehyde dehydrogenase 1 is a putative marker for cancer stem cells in head and neck squamous cancer. Biochem Biophys Res Commun.

[b7] Clarke MF, Dick JE, Dirks PB, Eaves CJ, Jamieson CH, Jones DL, Visvader J, Weissman IL, Wahl GM (2006). Cancer stem cells – perspectives on current status and future directions: AACR Workshop on cancer stem cells. Cancer Res.

[b8] Collins AT, Berry PA, Hyde C, Stower MJ, Maitland NJ (2005). Prospective identification of tumorigenic prostate cancer stem cells. Cancer Res.

[b9] Dekel B, Metsuyanim S, Schmidt-Ott KM, Fridman E, Jacob-Hirsch J, Simon A, Pinthus J, Mor Y, Barasch J, Amariglio N (2006). Multiple imprinted and stemness genes provide a link between normal and tumor progenitor cells of the developing human kidney. Cancer Res.

[b10] Dylla SJ, Beviglia L, Park IK, Chartier C, Raval J, Ngan L, Pickell K, Aguilar J, Lazetic S, Smith-Berdan S (2008). Colorectal cancer stem cells are enriched in xenogeneic tumors following chemotherapy. PLoS ONE.

[b11] Evseenko D, Zhu Y, Schenke-Layland K, Kuo J, Latour B, Ge S, Scholes J, Dravid G, Li X, MacLellan WR (2010). Mapping the first stages of mesoderm commitment during differentiation of human embryonic stem cells. Proc Natl Acad Sci USA.

[b12] Geenen MM, Cardous-Ubbink MC, Kremer LC, van den Bos C, van der Pal HJ, Heinen RC, Jaspers MW, Koning CC, Oldenburger F, Langeveld NE (2007). Medical assessment of adverse health outcomes in long-term survivors of childhood cancer. JAMA.

[b13] Gillespie GKFaGY (2011). Cancer stem cells and pediatric solid tumors. Cancers.

[b14] Ginestier C, Hur MH, Charafe-Jauffret E, Monville F, Dutcher J, Brown M, Jacquemier J, Viens P, Kleer CG, Liu S (2007). ALDH1 is a marker of normal and malignant human mammary stem cells and a predictor of poor clinical outcome. Cell Stem Cell.

[b15] Green DM, Beckwith JB, Breslow NE, Faria P, Moksness J, Finklestein JZ, Grundy P, Thomas PR, Kim T, Shochat S (1994). Treatment of children with stages II to IV anaplastic Wilms' tumor: a report from the National Wilms' Tumor Study Group. J Clin Oncol.

[b16] Green DM, Breslow NE, Evans I, Moksness J, D'Angio GJ (1996). Treatment of children with stage IV favorable histology Wilms tumor: a report from the National Wilms Tumor Study Group. Med Pediatr Oncol.

[b17] Gregory PA, Bert AG, Paterson EL, Barry SC, Tsykin A, Farshid G, Vadas MA, Khew-Goodall Y, Goodall GJ (2008). The miR-200 family and miR-205 regulate epithelial to mesenchymal transition by targeting ZEB1 and SIP1. Nat Cell Biol.

[b18] Grille SJ, Bellacosa A, Upson J, Klein-Szanto AJ, van Roy F, Lee-Kwon W, Donowitz M, Tsichlis PN, Larue L (2003). The protein kinase Akt induces epithelial mesenchymal transition and promotes enhanced motility and invasiveness of squamous cell carcinoma lines. Cancer Res.

[b19] Hu Q, Gao F, Tian W, Ruteshouser EC, Wang Y, Lazar A, Stewart J, Strong LC, Behringer RR, Huff V (2011). Wt1 ablation and Igf2 upregulation in mice result in Wilms tumors with elevated ERK1/2 phosphorylation. J Clin Invest.

[b20] Iliopoulos D, Lindahl-Allen M, Polytarchou C, Hirsch HA, Tsichlis PN, Struhl K (2010). Loss of miR-200 inhibition of Suz12 leads to polycomb-mediated repression required for the formation and maintenance of cancer stem cells. Mol Cell.

[b21] Iliopoulos D, Polytarchou C, Hatziapostolou M, Kottakis F, Maroulakou IG, Struhl K, Tsichlis PN (2009). MicroRNAs differentially regulated by Akt isoforms control EMT and stem cell renewal in cancer cells. Sci Signal.

[b22] James RG, Kamei CN, Wang Q, Jiang R, Schultheiss TM (2006). Odd-skipped related 1 is required for development of the metanephric kidney and regulates formation and differentiation of kidney precursor cells. Development.

[b23] Jensen M, Berthold F (2007). Targeting the neural cell adhesion molecule in cancer. Cancer Lett.

[b24] Jin L, Hope KJ, Zhai Q, Smadja-Joffe F, Dick JE (2006). Targeting of CD44 eradicates human acute myeloid leukemic stem cells. Nat Med.

[b25] Kashyap V, Rezende NC, Scotland KB, Shaffer SM, Persson JL, Gudas LJ, Mongan NP (2009). Regulation of stem cell pluripotency and differentiation involves a mutual regulatory circuit of the NANOG, OCT4, and SOX2 pluripotency transcription factors with polycomb repressive complexes and stem cell microRNAs. Stem Cells Dev.

[b26] Kaste SC, Dome JS, Babyn PS, Graf NM, Grundy P, Godzinski J, Levitt GA, Jenkinson H (2008). Wilms tumour: prognostic factors, staging, therapy and late effects. Pediatr Radiol.

[b27] Kobayashi A, Valerius MT, Mugford JW, Carroll TJ, Self M, Oliver G, McMahon AP (2008). Six2 defines and regulates a multipotent self-renewing nephron progenitor population throughout mammalian kidney development. Cell Stem Cell.

[b28] Kong D, Banerjee S, Ahmad A, Li Y, Wang Z, Sethi S, Sarkar FH (2011). Epithelial to mesenchymal transition is mechanistically linked with stem cell signatures in prostate cancer cells. PLoS ONE.

[b29] Kozaki K, Imoto I, Mogi S, Omura K, Inazawa J (2008). Exploration of tumor-suppressive microRNAs silenced by DNA hypermethylation in oral cancer. Cancer Res.

[b30] Lapidot T, Sirard C, Vormoor J, Murdoch B, Hoang T, Caceres-Cortes J, Minden M, Paterson B, Caligiuri MA, Dick JE (1994). A cell initiating human acute myeloid leukaemia after transplantation into SCID mice. Nature.

[b31] Li F, Tiede B, Massague J, Kang Y (2007). Beyond tumorigenesis: cancer stem cells in metastasis. Cell Res.

[b32] Li Y, VandenBoom TG, Kong D, Wang Z, Ali S, Philip PA, Sarkar FH (2009). Up-regulation of miR-200 and let-7 by natural agents leads to the reversal of epithelial-to-mesenchymal transition in gemcitabine-resistant pancreatic cancer cells. Cancer Res.

[b33] Ma S, Chan KW, Lee TK, Tang KH, Wo JY, Zheng BJ, Guan XY (2008). Aldehyde dehydrogenase discriminates the CD133 liver cancer stem cell populations. Mol Cancer Res.

[b34] Maiti S, Alam R, Amos CI, Huff V (2000). Frequent association of beta-catenin and WT1 mutations in Wilms tumors. Cancer Res.

[b35] Mani SA, Guo W, Liao MJ, Eaton EN, Ayyanan A, Zhou AY, Brooks M, Reinhard F, Zhang CC, Shipitsin M (2008). The epithelial-mesenchymal transition generates cells with properties of stem cells. Cell.

[b36] Martelli AM, Evangelisti C, Chiarini F, Grimaldi C, Cappellini A, Ognibene A, McCubrey JA (2010). The emerging role of the phosphatidylinositol 3-kinase/Akt/mammalian target of rapamycin signaling network in normal myelopoiesis and leukemogenesis. Biochim Biophys Acta.

[b37] Metsuyanim S, Harari-Steinberg O, Buzhor E, Omer D, Pode-Shakked N, Ben-Hur H, Halperin R, Schneider D, Dekel B (2009). Expression of stem cell markers in the human fetal kidney. PLoS ONE.

[b38] Metsuyanim S, Pode-Shakked N, Schmidt-Ott KM, Keshet G, Rechavi G, Blumental D, Dekel B (2008). Accumulation of malignant renal stem cells is associated with epigenetic changes in normal renal progenitor genes. Stem Cells.

[b39] Monticone M, Biollo E, Fabiano A, Fabbi M, Daga A, Romeo F, Maffei M, Melotti A, Giaretti W, Corte G (2009). z-Leucinyl-leucinyl-norleucinal induces apoptosis of human glioblastoma tumor-initiating cells by proteasome inhibition and mitotic arrest response. Mol Cancer Res.

[b40] Moss TJ, Strauss LC, Das L, Feig SA (1989). Secondary leukemia following successful treatment of Wilms' tumor. Am J Pediatr Hematol Oncol.

[b41] Mugford JW, Sipila P, McMahon JA, McMahon AP (2008). Osr1 expression demarcates a multi-potent population of intermediate mesoderm that undergoes progressive restriction to an Osr1-dependent nephron progenitor compartment within the mammalian kidney. Dev Biol.

[b42] Naor D, Sionov RV, Ish-Shalom D (1997). CD44: structure, function, and association with the malignant process. Adv Cancer Res.

[b43] O'Brien CA, Pollett A, Gallinger S, Dick JE (2007). A human colon cancer cell capable of initiating tumour growth in immunodeficient mice. Nature.

[b44] Oliver JA, Barasch J, Yang J, Herzlinger D, Al-Awqati Q (2002). Metanephric mesenchyme contains embryonic renal stem cells. Am J Physiol Renal Physiol.

[b45] Ovcharenko D, Kelnar K, Johnson C, Leng N, Brown D (2007). Genome-scale microRNA and small interfering RNA screens identify small RNA modulators of TRAIL-induced apoptosis pathway. Cancer Res.

[b46] Paterson EL, Kolesnikoff N, Gregory PA, Bert AG, Khew-Goodall Y, Goodall GJ (2008). The microRNA-200 family regulates epithelial to mesenchymal transition. Sci World J.

[b47] Pearce DJ, Taussig D, Simpson C, Allen K, Rohatiner AZ, Lister TA, Bonnet D (2005). Characterization of cells with a high aldehyde dehydrogenase activity from cord blood and acute myeloid leukemia samples. Stem Cells.

[b48] Peter ME (2009). Let-7 and miR-200 microRNAs: guardians against pluripotency and cancer progression. Cell Cycle.

[b49] Pinkerton CR, Groot-Loonen JJ, Morris-Jones PH, Pritchard J (1991). Response rates in relapsed Wilms' tumor. A need for new effective agents. Cancer.

[b50] Pode-Shakked N, Metsuyanim S, Rom-Gross E, Mor Y, Fridman E, Goldstein I, Amariglio N, Rechavi G, Keshet G, Dekel B (2009). Developmental tumourigenesis: NCAM as a putative marker for the malignant renal stem/progenitor cell population. J Cell Mol Med.

[b51] Quintana E, Shackleton M, Sabel MS, Fullen DR, Johnson TM, Morrison SJ (2008). Efficient tumour formation by single human melanoma cells. Nature.

[b52] Reinhard H, Schmidt A, Furtwangler R, Leuschner I, Rube C, Von Schweinitz D, Zoubek A, Niggli F, Graf N (2008). Outcome of relapses of nephroblastoma in patients registered in the SIOP/GPOH trials and studies. Oncology Rep.

[b53] Reya T, Morrison SJ, Clarke MF, Weissman IL (2001). Stem cells, cancer, and cancer stem cells. Nature.

[b54] Ricart AD (2011). Immunoconjugates against solid tumors: mind the gap. Clin Pharmacol Ther.

[b55] Rivera MN, Haber DA (2005). Wilms' tumour: connecting tumorigenesis and organ development in the kidney. Nat Rev.

[b56] Rivera MN, Kim WJ, Wells J, Driscoll DR, Brannigan BW, Han M, Kim JC, Feinberg AP, Gerald WL, Vargas SO (2007). An X chromosome gene, WTX, is commonly inactivated in Wilms tumor. Science (New York, NY).

[b57] Ronconi E, Sagrinati C, Angelotti ML, Lazzeri E, Mazzinghi B, Ballerini L, Parente E, Becherucci F, Gacci M, Carini M (2009). Regeneration of glomerular podocytes by human renal progenitors. J Am Soc Nephrol.

[b58] Roth J, Blaha I, Bitter-Suermann D, Heitz PU (1988a). Blastemal cells of nephroblastomatosis complex share an onco-developmental antigen with embryonic kidney and Wilms' tumor. An immunohistochemical study on polysialic acid distribution. Am J Pathol.

[b59] Roth J, Zuber C, Wagner P, Blaha I, Bitter-Suermann D, Heitz PU (1988b). Presence of the long chain form of polysialic acid of the neural cell adhesion molecule in Wilms' tumor. Identification of a cell adhesion molecule as an oncodevelopmental antigen and implications for tumor histogenesis. Am J Pathol.

[b60] Schatton T, Murphy GF, Frank NY, Yamaura K, Waaga-Gasser AM, Gasser M, Zhan Q, Jordan S, Duncan LM, Weishaupt C (2008). Identification of cells initiating human melanomas. Nature.

[b61] Schmelzer E, Wauthier E, Reid LM (2006). The phenotypes of pluripotent human hepatic progenitors. Stem Cells.

[b62] Schnabel CA, Godin RE, Cleary ML (2003). Pbx1 regulates nephrogenesis and ureteric branching in the developing kidney. Dev Biol.

[b63] Self M, Lagutin OV, Bowling B, Hendrix J, Cai Y, Dressler GR, Oliver G (2006). Six2 is required for suppression of nephrogenesis and progenitor renewal in the developing kidney. EMBO J.

[b64] Silva IA, Bai S, McLean K, Yang K, Griffith K, Thomas D, Ginestier C, Johnston C, Kueck A, Reynolds RK (2011). Aldehyde dehydrogenase in combination with CD133 defines angiogenic ovarian cancer stem cells that portend poor patient survival. Cancer Res.

[b65] Singh SK, Clarke ID, Terasaki M, Bonn VE, Hawkins C, Squire J, Dirks PB (2003). Identification of a cancer stem cell in human brain tumors. Cancer Res.

[b66] Singh SK, Hawkins C, Clarke ID, Squire JA, Bayani J, Hide T, Henkelman RM, Cusimano MD, Dirks PB (2004). Identification of human brain tumour initiating cells. Nature.

[b67] Sparano JA, Beckwith JB, Mitsudo S, Wiernik PH (1991). Complete remission in refractory anaplastic adult Wilms' tumor treated with cisplatin and etoposide. Cancer.

[b68] Takahashi M, Yang YX, Lavery TT, Furge KA, Williams BO, Tretiakova M, Montag A, Vogelzang NJ, Re GG, Garvin AJ, Söderhäll S, Kagawa S, Hazel-Martin D, Nordenskjold A, Teh BT (2002). Gene expression profiling of favorable histology Wilms' tumors and its correlation with clinical features. Cancer Res.

[b69] Tanei T, Morimoto K, Shimazu K, Kim SJ, Tanji Y, Taguchi T, Tamaki Y, Noguchi S (2009). Association of breast cancer stem cells identified by aldehyde dehydrogenase 1 expression with resistance to sequential Paclitaxel and epirubicin-based chemotherapy for breast cancers. Clin Cancer Res.

[b70] Thomas S, Thomas M, Wincker P, Babarit C, Xu P, Speer MC, Munnich A, Lyonnet S, Vekemans M, Etchevers HC (2008). Human neural crest cells display molecular and phenotypic hallmarks of stem cells. Hum Mol Genet.

[b71] Tsokos M, Scarpa S, Ross RA, Triche TJ (1987). Differentiation of human neuroblastoma recapitulates neural crest development. Study of morphology, neurotransmitter enzymes, and extracellular matrix proteins. Am J Pathol.

[b72] Vane D, King DR, Boles ET (1984). Secondary thyroid neoplasms in pediatric cancer patients: increased risk with improved survival. J Pediatr Surg.

[b73] Wang FE, Zhang C, Maminishkis A, Dong L, Zhi C, Li R, Zhao J, Majerciak V, Gaur AB, Chen S (2010). MicroRNA-204/211 alters epithelial physiology. FASEB J.

[b74] Wang X, Luo H, Chen H, Duguid W, Wu J (1998). Role of proteasomes in T cell activation and proliferation. J Immunol.

[b75] Wen JG, van Steenbrugge GJ, Egeler RM, Nijman RM (1997). Progress of fundamental research in Wilms' tumor. Urol Res.

[b76] Wittmann S, Wunder C, Zirn B, Furtwangler R, Wegert J, Graf N, Gessler M (2008). New prognostic markers revealed by evaluation of genes correlated with clinical parameters in Wilms tumors. Genes Chromosomes Cancer.

