# Supplementary material for: The isolation and characterization of renal cancer initiating cells from human Wilms' tumour xenografts unveils new therapeutic targets
Source: EMBO Mol Med. 2012 Dec 13;5(1):18–37. doi: 10.1002/emmm.201201516 (PMC3569651; doi:10.1002/emmm.201201516)
Supplement: Supplementary file 1 [file emmm0005-0018-SD1.pdf]

EMBO Molecular Medicine

Manuscript EMM-2012-01516

The isolation and characterization of renal cancer initiating cells from human Wilms' tumor xenografts unveils new therapeutic targets

Naomi Pode-Shakked, Rachel Shukrun, Michal Mark-Danieli, Peter Tsvetkov, Sarit Bahar, Sara Pri-Chen, Ronald S. Goldstein, Eithan Rom-Gross, Yoram Mor, Edward Fridman, Karen Meir, Amos Simon, Marcus Magister, Naftali Kaminski, Victor S. Goldmacher, Orit Harari-Steinberg, Benjamin Dekel

Corresponding author: Benjamin Dekel, The Pediatric Stem Cell Research Institute, Edmond & Lily Safra Children's Hospital, Sheba Center for Regenerative Medicine, Sheba Medical Center, Israel.

Review timeline:

Submission date:	29 April 2012
Editorial Decision:	06 June 2012
Revision received:	14 September 2012
Editorial Decision:	12 October 2012
Revision received:	22 October 2012
Accepted:	23 October 2012

Transaction Report:

(Note: With the exception of the correction of typographical or spelling errors that could be a source of ambiguity, letters and reports are not edited. The original formatting of letters and referee reports may not be reflected in this compilation.)

1st Editorial Decision 06 June 2012

Thank you for the submission of your manuscript to EMBO Molecular Medicine. We have now heard back from two out of the three referees whom we asked to evaluate your manuscript. As both referees share a similar overall positive opinion about the manuscript, and as the last referee is already late, we thought of letting you know of our pre-decision. Please note that in case where the third referee would have major serious issues, the decision may change.

As you will see from the comments below, both referees find the study important. However they do raise some concerns particularly regarding quantitation analyses often missing or inappropriate. In addition, Referee #1 wonders about the methylation status of the imprinted IGF2 locus. But more importantly, Referee #2 would like to see if transplanted tumors in a different mouse model would give similar results.

Given the balance of these evaluations, we feel that we can consider a revision of your manuscript if you can address all the issues that have been raised within the space and time constraints outlined below. Please note that it is EMBO Molecular Medicine policy to allow only a single round of revision and that, as acceptance or rejection of the manuscript will depend on another round of review, your responses should be as complete as possible.

We will forward you the last report, together with our formal decision, as soon as it will be available.

Revised manuscripts should be submitted within three months of a request for revision; they will otherwise be treated as new submissions, except under exceptional circumstances in which a short extension is obtained from the editor. Also, the length of the revised manuscript may not exceed 60,000 characters (including spaces) and, including figures, the paper must ultimately fit onto optimally ten pages of the journal. You may consider including any peripheral data (but not methods in their entirety) in the form of Supplementary information.

I look forward to seeing a revised form of your manuscript as soon as possible.

Yours sincerely,

Editor
EMBO Molecular Medicine

***** Reviewer's comments *****

Referee #1:

Wilms' tumours have always been a model for the role of disturbed development in tumourigenesis. It is therefore surprising that the cancer stem cell hypothesis so far has gotten very little attention. In this manuscript, Pode-Shakked et al make some long overdue and important contributions to this field.

First, they described an optimized protocol for the generation of WT xenografts using tumour fragments, giving 80% uptake. This is a very impressive improvement over existing protocols and will greatly enhance the possibilities for drug testing. The Xns could be propagated with decreasing number of cells which show an increase in early mesenchymal markers like SIX2 and OSR1, suggesting enrichment for cancer stem cells. The putatives stem cell marker NCAM was found to be enriched in the p-Xns, whereas CD133 was absent, suggesting NCAM is an important marker for these CRC. Indeed, as few as 500 NCAM+ cells was found to be sufficient for Xn take, something further approved to just 200 cells when using NCAM+ ALDH1+ cells. These cells fully recapitulate the histology of the parental tumours. Protein and miRNA analysis are used to further describe these cells. As the CRC hypothesis also states that it is these cells that need to be specifically targeted in therapy, the authors show the conventional first line therapy does not lead to a reduction of couble positive cells, whereas second line treatment (normally used for relapsing WT) does. As icing on the cake the authors show that NCAM antibody-drug conjugates can eradicate the Xns in vivo.

This is a very important manuscript. There is no doubt in my mind these NCAM+ALDH1+ cells are indeed Wilms' tumour CRCs, and as such this manuscript provides essential information for the origin and treatment of WTs. I only have minor comments.

1. The tumours are described as 'thriphasic favorable histology WT lacking mutations in b-catenin, WT1 and WTX'. It has always been my impression that these WT1 mutant (and by extension therefore b-catenin mutant) tumours are the favorable histology and are the stromal pre-dominant, whereas triphasic WT1/ beta-catenin wildtype tumours are non-favorable histology. Is this maybe a type in the manuscript?

2. As the WT1/b-catenin wildtype set of WT is now very well defined with respect to genetic aberrations, it would be interesting to know the methylation status of the imprinted 11p15 IGF2 locus, especially in the context of the 20% of tumours that did not take in the primary Xn.

3. As the authors point out, it is striking that the signal for NCAM and ALDH1 is not fully overlapping. The authors could maybe comment on the implications of the fact that increased RA metabolism would result in an increase of an secreted factor in their models for the role of NCAM and ALDH1 in these CRCs.

Referee #2 (Comments on Novelty/Model System):

This paper details the first study to address the existence of cancer initiating cells in pediatric WTs using in-vivo model. It represents an important advance in studying WT biology, since many kinds of kidney cancer, including adult RCC (renal cell carcinoma) and pediatric WT are known to lack animal models allowing tumor growth. Using primary xenograft assays authors identify NCAM+ tumor initiating cell population that can further be sub-fractionated using ALDH marker. The paper also describes interesting correlations between NCAM expression and expression of "stemness markers", as well as includes miRNA profiling and phosphoprotein profiling to implicate miR200 and let7 families of microRNAs as well as Akt phosphorilation as possible molecular mechanisms driving proliferation of malignant renal stem cells. In addition, authors provided data on treatment of WT bulk population with two types of chemotherapy and showed the reduction in NCAM+ALDH+ cell population specifically after etoposide plus cisplatin drug combination, which showed efficacy in the clinic. Finally, they used the cytotoxic antibody conjugate to target NCAM+ cells in vivo.

While the amount of data is compelling certain areas of this study raise concerns (described in detail in the remarks section) which need to be addressed before it can be accepted for publication

Referee #2 (Other Remarks):

Major concerns:

1. all experiments are done on xenografted tumors that had undergone adaptation to the mouse host and are composed mostly of NCAM+ cells, while their respective primary human counterparts had only minor subset of NCAM+ cells. Did authors try isogenic transplantation of primary human tumor cells into kidney capsule? Also the use of severely immuno-defficient mouse strains (NSG or Rag2-/gc DKO) for testing tumor initiating properties of human tumor cells should also be considered. Either of those models should be tried with at least one tumor to demonstrate that it behaves similarly to NOD/SCID subqutaneous injection model.

2. In almost all figures related to the immuno-histochemical analysis where expression of NCAM (Fig.1-5) in tumor and differentiated cells and/or stroma is correlated with the markers described in the text such as Ki67, WT1 and ALDH different fields of the same tissue are photographed for NCAM, Ki67, WT1 and ALDH stainings. It would critical to see the same fields and same magnification to draw respective conclusions. The panoramic view of tumor sections at lower mag would be also be very helpful.

3. Actual FACS analysis figures and gating trees that include lineage/mouse or human lineage/stroma cell gates are missing for most of the tumors from which cancer initiating cells were isolated using NCAM (tumors described in table 1) and NCMA/ALDH as described in table 2. Showing post sort populations used for injections described in both tables would also be helpful.

4.Addition of ALDH marker clearly separates further NCAM+ population into TSC and non TSC , however one would expect that NCAM+/ALDH+ cells would be more tumorigenic than bulk NCAM+ cells, but based on table 2 data (upper and bottom sections) there is insufficient number of cell dose titration and injections to see the increase in tumorigenicity with addition of ALDH marker. Do authors have more data or explanation for this outcome?

Minor Concerns:

Figure 1A shows that NCAM and Ki67 specifically localize in blastema compartment of p-WT Xn, at the same time no serial sections of the same tumor are presented. It would be helpful if authors could stain serial sections and photograph the corresponding fields to see the overlap of NCAM1 and Ki67, as well as WT1 and quantitate the areas of positive blastemavs positive non-blastema as well as the overlap.
Fig. 1F: Please average 2-3 representative blots for quantitation to acquire error bars or remove the quantitation...
Fig. 1G Please add error bars in np-WT Xn

Fig.3 Two fields for NCAM+ Xn are shown, one with NCAM+ cells and the other with NCAM- cells, which predominate. It is hard to judge about Ki67 and WT1 in relation to NCAM in a non serial section. The panoramic view of tumor section would be also helpful...Fig.3c NCAM- cells in the tumor might derive from differentiation of NCAM+ cells, but also possibly from normal stroma/epiphelia/glumeroloid bodies present in vicinity to invading tumor cells. Could authors provide the FACS data on how they sorted the primary tumor and if they considered removal NCAM- cells of normal tissue origin.
Fig.4. Please, make sure that all axes have the correct information on what is presented on the plot, especially see Fig4 FACS plots, should it be NCAM instead of isotype in Aa, Ba, Ca, Cd? Error bars are missing in figure 4Ce, and p value not calculated. Fig 4C, did the authors analyze NCAM+ALDHA+ vs NCAM+ALDH1-, similar to analyses in A and B? Again, the sections are non serial and it looks like in NCAM+CD44- Xn NCAM expression is pretty low judging by the picture provided
Fig. 5 The use of fetal kidney to localize NCAM expression is very important for elucidation of developmental steps, if possible authors should consider staining young adult human kidneys to demonstrate in which compartments NCAM is expressed since capping mesenchyme is specific to developing embryo and is not present in the kidneys of new-borns or adult human organisms.
Figure 6B Western blot should be repeated on one gel: it looks like the AktSer473 and a-tubulin are compiled from several blots, all the lanes should be run on same gel.
Fig.7 Scale is missing on several panels
Figure 8. The effect of targeted antibodies treatment on the tumor burden is very encouraging, however in all graphs either control or experimental curves are lacking error bars, assuming multiple mice were used in each treatment group authors need to indicate proper distribution of tumor size between different animals in the same group. In addition, why authors are using tumor size percent, which corresponds to % of largest tumor in the group to quantitate results? Could the authors re-quantitate results using a more traditional way of assessing tumor size in mm3, or at least provide the data on average tumor size for each group in supplemental with the explanation of the rational for using this technique. What is the FACS profile of remaining or resistant tumors, did authors ever observe NCAM- cells ever become NCAM+ cells?

1st Revision - authors' response 14 September 2012

Referee # 1

Minor Revisions

1. *The tumours are described as 'thriphasic favorable histology WT lacking mutations in b-catenin, WT1 and WTX'. It has always been my impression that these WT1 mutant (and by extension therefore b-catenin mutant) tumours are the favorable histology and are the stromal pre-dominant, whereas triphasic WT1/ beta-catenin wild type tumours are non-favorable histology. Is this maybe a type in the manuscript*

We thank the referee for the comment. The histological classification of Wilms' tumor is defined by both the NWTS and SIOP studies. According to the NWTS histological classification most WTs have a favorable histology; however, about 5% have an unfavorable histology with anaplastic changes. Anaplasia is the only criterion for assigning a WT as having "unfavorable histology" regardless of its components or mutational status. Each of the three cellular components of WT can exhibit focal or diffuse anaplasia which has been characterized as tumors containing larger nuclei, increased nuclear chromatin content, and polyploid mitotic figures. Diffuse anaplastic changes generally predict a poor outcome. In addition, Wilms' tumors with anaplastic changes are more resistant to chemotherapy and therefore require more aggressive treatment. In this study, only WTs without anaplastic changes (favorable histology) that contained all three major WT components (i.e. blastema, epithelia, stroma) and lacked mutations in β-catenin, WT1 and WTX, were used.

2. *As the WT1/b-catenin wild type set of WT is now very well defined with respect to genetic aberrations, it would be interesting to know the methylation status of the imprinted 11p15 IGF2 locus, especially in the context of the 20% of tumours that did not take in the primary Xn.*

We thank the referee for the comment regarding the methylation status of the imprinted 11p15 IGF2 locus. In this work we did not focus on epigenetic changes in WT though we find it to be an important and compelling issue as well. We aim in futuristic experiments to study the methylation pattern and its relevance to WT xenograft formation and propagation.

3. As the authors point out, it is striking that the signal for NCAM and ALDH1 is not fully overlapping. The authors could maybe comment on the implications of the fact that increased RA metabolism would result in an increase of an secreted factor in their models for the role of NCAM and ALDH1 in these CRCs.

We thank the referee for the comment. We find the topic to be extremely interesting and we are currently examining possible implications of RA metabolism in Wilms' tumor CSCs.

Referee #2

Major Revisions

1. All experiments are done on xenografted tumors that had undergone adaptation to the mouse host and are composed mostly of NCAM^{+} cells, while their respective primary human counterparts had only minor subset of NCAM^{+} cells. Did authors try isogenic transplantation of primary human tumor cells into kidney capsule? Also the use of severely immunodefficient mouse strains (NSG or Rag2-/gc DKO) for testing tumor initiating properties of human tumor cells should also be considered. Either of those models should be tried with at least one tumor to demonstrate that it behaves similarly to NOD/SCID subcutaneous injection model.

Thank you for this comment. We want to emphasize that we have analysed two strains of immunodeficient mice for WT initiation and propagation, e.g., NOD/SCID (T-B-) as well as SCID/Biege (T-B-NK-), and no differences whatsoever were detected in growth patterns (See Supporting Information Fig S1). Moreover, in accordance with the referee's comment we have now performed a set of limiting dilution experiments (in addition to our data in NOD/SCID mice) in which immunosorted WT cells were injected into 14 NOGs. Similar to NOD/SCID experiments, NCAM^{+}ALDH^{+} cells were we found to be the CIC fraction in NOGs, further ascertaining the results in an additional immunodeficient mouse model. These data are presented in Table 4.

2. In almost all figures related to the immuno-histochemical analysis where expression of NCAM (Fig.1-5) in tumor and differentiated cells and/or stroma is correlated with the markers described in the text such as Ki67, WT1 and ALDH different fields of the same tissue are photographed for NCAM, Ki67, WT1 and ALDH stainings. It would critical to see the same fields and same magnification to draw respective conclusions. The panoramic view of tumor sections at lower mag would be also be very helpful.

In accordance with the referee's comment we are now showing all of the immuno-histochemical data in serial sections showing consistent fields (See Figs. 1-3, and Supporting Information Figs. S2 and S4). In addition, as requested we have added a panoramic view of all tumour sections (represented in supp. Fig. S2D).

3. Actual FACS analysis figures and gating trees that include lineage/mouse or human lineage/stroma cell gates are missing for most of the tumors from which cancer initiating cells were isolated using NCAM (tumors described in table 1) and NCMA/ALDH as described in table 2. Showing post sort populations used for injections described in both tables would also be helpful.

We have added the FACS analysis of H2K^{+} cells sorted WT Xn showing lack of mouse cell contamination, (see Fig. S3A). In order to demonstrate that only NCAM^{+} ALDH$^{+/-}$ cells were injected into the mice, we also show the gates from which the cells were sorted (see Supporting Information Fig. S5A). In addition, we have added FACS analysis of post-sorted populations for NCAM^{+} cells, as shown in Supporting Information Fig. S3B. Since technical problems do not allow us to show post-sorted data regarding ALDH^{+} (cells after sorting cannot be maintained viable in the

ALDH kit buffer), we added q-RT-PCR of ALDH1 post-sorting, showing no expression in the negative fractions (Supporting Information Fig. S5B).

4. Addition of ALDH marker clearly separates further NCAM$^+$ population into TSC and non TSC, however one would expect that NCAM$^+$/ALDH$^+$ cells would be more tumorigenic than bulk NCAM$^+$ cells, but based on table 2 data (upper and bottom sections) there is insufficient number of cell dose titration and injections to see the increase in tumourigenicity with addition of ALDH marker. Do authors have more data or explanation for this outcome?

To increase the robustness of our results we have now performed additional LD xenotransplantation experiments and include statistical power (summarized in Table 3). In addition, new self-renewal experiments with sorted NCAM$^+$ALDH$^+$ cells injected into secondary hosts were carried out (summarized in Table 4). These new experiments unequivocally show that NCAM$^+$ALDH$^+$ and not NCAM$^+$ALDH$^-$ is a tumour initiating as well as self-renewing cell fraction.

Minor Revisions

Figure 1

Fig. 1A: The figure shows that NCAM and Ki67 specifically localize in blastema compartment of p-WT Xn, at the same time no serial sections of the same tumour are presented. It would be helpful if authors could stain serial sections and photograph the corresponding fields to see the overlap of NCAM1 and Ki67, as well as WT1 and quantitate the areas of positive blastema vs. positive non-blastema as well as the overlap.

All immuno-histochemical staining in the figure are now presented in serial sections. In addition, we have added a quantitative analysis comparing between blastema and non-blastema tissues showing the difference in ki67 staining (Fig. 1B).

Fig. 1F: Please average 2-3 representative blots for quantitation to acquire error bars or remove the quantitation.

Quantitation has been removed.

Fig. 1G Please add error bars in np-WT Xn

We appreciate the referee's comment. However, error bars cannot be added because the analysis was preformed on two primary Wilms' tumours.

Figure 3

Fig.3 Two fields for NCAM$^+$ Xn are shown, one with NCAM$^+$ cells and the other with NCAM$^-$ cells, which predominate. It is hard to judge about Ki67 and WT1 in relation to NCAM in a non serial section. The panoramic view of tumour section would be also helpful.

All immuno-histochemical staining in the figure is now shown in serial sections so as to afford clearer presentation of data. In addition, we have added a panoramic view of the tumours (Supporting Information Fig. S2D).

Fig.3c NCAM$^-$ cells in the tumour might derive from differentiation of NCAM$^+$ cells, but also possibly from normal stroma/epiphelia/glumeroloid bodies present in vicinity to invading tumour cells. Could authors provide the FACS data on how they sorted the primary tumour and if they considered removal NCAM$^-$ cells of normal tissue origin.

We thank the referee for this comment.

a. All the tumors in our experiments were received from trained pathologists who specifically gave us tissue fragments from the tumor core (far from normal renal tissue).
b. WT tends to be encapsulated and therefore normal cell invasion is very unlikely.

c. FACS sorting data verifying lack of NCAM- cells injected into mice receiving the positive fraction (NCAM+ALDH+) (Supporting Information Fig S5A), in addition to the post sort FACS analysis, showing maximal sorting purity (Supporting Information Fig S3B).

d. We excluded the option that NCAM- mice cells contaminated the tumor by using H2K staining, the FACS analysis is shown in Supporting Information Fig. S3A.

Figure 4
Please, make sure that all axes have the correct information on what is presented on the plot, especially see Fig4 FACS plots, should it be NCAM instead of isotype in Aa, Ba, Ca, Cd?

Labeling of the axes has been corrected (Fig 3A).

Error bars are missing in Fig. 4Ce, and p value not calculated.

Error bars were added to the figure and P value was calculated (see Fig 3E). .

Fig 4C, did the authors analyze $NCAM^+ALDHA^+$ vs. $NCAM^+ALDH1^-$, similar to analyses in A and B?

We thank the referee for this comment; however, since no tumors developed from the $NCAM^+$ $ALDH1^-$ fraction, there is no option to perform the described analysis.

Again, the sections are non serial and it looks like in $NCAM^+$ $CD44^-$ Xn NCAM expression is pretty low judging by the picture provided.

All immuno-histochemical stainings in the figure are now presented in serial sections. New pictures of $NCAM^+CD44^-$ sections were taken (Fig. S4B).

Figure 5
The use of fetal kidney to localize NCAM expression is very important for elucidation of developmental steps, if possible authors should consider staining young adult human kidneys to demonstrate in which compartments NCAM is expressed since capping mesenchyme is specific to developing embryo and is not present in the kidneys of new-borns or adult human organisms.

We thank the referee for this comment. Young adult human kidneys are not available to us at the moment. We aim to address this in futuristic experiments.

Figure 6B
Figure 6B Western blot should be repeated on one gel: it looks like the AktSer473 and a-tubulin are compiled from several blots; all the lanes should be run on same gel.

We thank the referee for this comment. As custom, all our lanes were run on the same gel. However, due to irrelevant samples in the gel, we edited the blots. Attached please find the original gel blots.

Figure 7
Figure 7 Scale is missing on several panels

The bar graphs of Figs. 7C.a and 7C.b are analyses of cell survival performed in parallel to respective FACS analysis on the right. We have now clarified this issue in the figure legend and the statistical analysis of this correlation is depicted in Supporting Information Fig. S7.

Figure 8.
The effect of targeted antibodies treatment on the tumour burden is very encouraging, however in all graphs either control or experimental curves are lacking error bars, assuming multiple mice were used in each treatment group authors need to indicate proper distribution of tumour size between different animals in the same group. In addition, why authors are using tumour size percent, which corresponds to % of largest tumour in the group to quantitate results? Could the authors re-quantitate results using a more traditional way of assessing tumour size in mm3, or at least provide the data on average tumour size for each group in supplemental with the explanation

of the rational for using this technique. What is the FACS profile of remaining or resistant tumours, did authors ever observe NCAM⁻ cells ever become NCAM⁺ cells?

We increased the statistical sample size for all in vivo treatment experiments and presented the tumor size in mm^3, as requested (new Fig. 5). We have also added the FACS analysis of the resistant tumors in Supporting Information Fig. S8.

Altogether, the new *in vivo* treatment data added to the older experiments are extremely robust.

Referee #3

Major Revisions

1. Comments on Novelty/Model System:

The major limitation and weakness of this study is that the authors directly relate the results from cells isolated mouse xenografts to human primary tumours. This starts with the title which implies that the cells being characterized and eradicated are directly from a renal tumour. The title needs to clearly state that the isolation and characterization is from xenografts of human renal tumours. The first sentence of the discussion directly states that a TIC/CSC has been identified in Wilms' tumour. This is incorrect. The TIC/CSC was identified in xenografts.

This is not a trivial issue. As stated by the authors, xenograft take is only ~10% when they are generated from single cell suspensions, even though good number of WTs selected in this study contains 10-70% of NCAM⁺ cells. Even if we agree NCAM also marks cancer stem cells (CSC) in the primary tumours, it is reasonable to hypothesize that the biology and behaviour of the CSCs in the primary tumour are different from those of the TICs in the WT xenografts. So the identity or knowledge of the WT TICs is true in p-WT xenografts but cannot be simply extended to the WT CSCs in primary tumours before being proved. Similarly, the high efficacy of the studied drug cannot be over-interpreted given the fact that only the xenograft model derived from WT xenograft TICs were tested and the non-propagatable WT could not be tested due to a dramatically reduced number of NCAM⁺ cells in the xenograft. To avoid giving an impression the WT TIC identified in this study is the same thing as a genera WT CSC, minimal use of CSC in this manuscript is suggested and all limitations mentioned above should be well discussed.
Considering the novelty of this study, it is still significant progress in the field of WT research. The findings may provide new insight into the cell of origin in WT and potentially lead to new biomarker-based therapy. The authors just need to be very clear about the xenograft origin of the cells they characterize and therapeutically treat and they need to confine their inferences to xenografts with the possibility that a similar type of cell is present in primary tumours.

We thank the referee for the comment; we have changed the title of the manuscript and the wording along all parts of the text so as to emphasize the model human xenograft system we have used.
It is crucial to understand that tumorigenic favourable histology WT cell lines are not available, in addition to the fact that cells within WT, as with other pediatric solid tumours, are less accessible (compared to adult carcinomas). Therefore, the Xn model system is the only model that allows *in vivo* studies of WT at the single cell level at the moment.

Minor Revisions

Figure 1
About the figure:
a) Label c for Fig 1D is missing and error bar for Fig 1G (grey column) should be placed.
b) White arrow stated in the legend (page 35 line 9) is not shown.
c) In Fig 1G, the label should be p-WT and np-WT instead of p-WT Xn and np WT Xn.

The labelling and error bars have been added to the Figure and the label in Fig 1G has been corrected (Fig 1E and 1H).

About the legend:
a) page 35 line 7, change an to and
b) line 20, change page to PAGE
c) line 29, change upper to left

Recommended changes have been made.

About the results:
a) page 5 line 11, indicate the patient code for the 4 WT sources so that readers know which tumours you talked about in a big table 1
b) page 6 line 9, indicate patient code for the one p-WT
c) page 6 line 15, correct sentence
d) page 6 line 17, at this point, you have not defined markers for WT Stem/progenitor cells, this conclusion cannot be inferred.

Recommended changes have been made.

Figure 2
About the figure:
a) Fig 2B, the NCAM column seems to have different magnification from the rest. If so, please indicate in the legend.

All immuno-histochemical stainings in the figure are now presented in serial sections in consistent magnification.

About the legend:
a) page 36 line 8, should have different magnifications for the upper and lower panels of Fig 2A

Recommended change has been made.

Figure 3
About the figure:
a) Fig 3E (b): Tubulin control lane 4 does not match other two 4th lanes.

The amount of protein in all samples was similar, as seen in Fig. 2Eb.

About the legend:
a) page 37 line 11, change WO14 to W014
About the results:
b) The data for NF-kB is presented in the figure but not interpreted.

Recommended changes have been made.

Figure 4
About the figure:
a) Fig 4A: panel labels c and d should be switched if you want to keep the legend same.

Recommended change has been made (Supporting Information Fig. S4)

b) Fig 4B(b): realignment of lane number and the gel.
c) Fig 4A(a) and Fig 4B(a) and Fig 4C(a): wrong label for Y axis.
d) Fig 4C(d): no label for Y axis.
e) Fig 4C(e): error bars missing and statistical analysis not done for colonies/5000 cells.

Recommended changes have been made (Fig. 3 and Supporting Information Fig. S4).

About the legend:
a) page 37 line 22: interpretation of the data is not appropriate. Some changes are obvious and significant.
About the results:
b) page 8 line 27-28: interpretation of Fig 4A(c) data is not appropriate.

Fig legend and results have been modified according to the referee's comments.

Figure 6
About the figure:
a) If you want to keep the legend same, you should add a panel label a for Fig 6C, change panel label D to b (under Fig 6C), change panel label E to label D.

Recommended changes have been made (Supporting Information Fig. S7B).

Figure 7
About the legend:
a) Page 40 lines 5 and 11: correct figure 30 in both places.

Recommended changes have been made.

Figure 8
About the figure:
a) Error bars missing in Fig 8A(a) (the 3rd graph), Fig 8A(b) (the 1st graph) and Fig 8B(a).

Recommended changes have been made (Fig. 5).

Table S2
The last row is incomplete.

The table in the manuscript is now complete (Supporting Information Table S2)
Citation and references
a) Years are not indicated in the following citations and corresponding references. 1) page 7 line 2; page 15 lines 15 and 26; page 16 line 27; page 23 for Pandit et al. Please check your references again to fill in possibly missing information.
b) The authors in reference Gillespie GK FaGY (page 29 line 20) are not spelled correctly.

Recommended changes have been made.

Experimental procedures

Page 20 paragraph "primary WT and WT Xn cell cultures": Correct the 1st sentence and fill in the growth factor in the end of the paragraph.

All changes recommended have been made in accordance with the referee's comments.

2nd Editorial Decision 12 October 2012

Thank you for the submission of your revised manuscript "Prospective isolation and characterization of renal cancer stem cells from human Wilms' tumor xenografts provides new therapeutic targets" to EMBO Molecular Medicine. We have now received the enclosed reports from the referees whom we asked to re-assess it. You will be glad to see that the reviewers are now globally supportive and

we can proceed with official acceptance of your manuscript pending the following minor changes:

- Please address the minor concerns raised by Reviewer #2 (see below).

- The description of all reported data that includes statistical testing must state the name of the statistical test used to generate error bars and P values, the number (n) of independent experiments underlying each data point (not replicate measures of one sample), and the actual P value for each test (not merely 'significant' or 'P < 0.05').

- In addition, we noted some minor concerns regarding the figures: Please ensure that the resolution of the line graphs is improved and that labels are readable also at lower magnification.

- The section For More Information should be used to list relevant web links for further consultation by our readers. Examples include patient associations, relevant databases, OMIM/proteins/genes links or author's websites. Since you listed on the SI, please either remove the For More Information section or include relevant links as described above.

Please submit your revised manuscript within two weeks. I look forward to seeing a revised form of your manuscript as soon as possible.

I look forward to reading a new revised version of your manuscript as soon as possible.

Yours sincerely,

Editor
EMBO Molecular Medicine

***** Reviewer's comments *****

Referee #2 (Comments on Novelty/Model System):

The revised version of the manuscript "Prospective isolation and characterization of renal cancer stem cells from human Wilms' tumor xenografts provides new therapeutic targets" by Pode-Shakked, N et. al is very much improved and is now suitable for publication. I believe this manuscript has very high medical impact delineating tumorigenic properties of Wilms' tumors at both cellular and molecular levels. In addition, authors provide in-vivo evidence that targeting Wilms' tumor stem cells with NCAM-conjugated antibody has therapeutic effect which opens the door for the development of more successful treatments for cancer patients in the clinic.
Few editing remarks are discussed below and should be considered in the final version of the manuscript.

Referee #2 (Other Remarks):

The manuscript has been significantly improved after authors addressed previous concerns and performed additional experiments. This work is now suitable for publication with few minor remarks:

Figure S1. (WT Xenograft system) should be considered as the main figure and be brought from supplemental section into the main text as it illustrates entire in-vivo experimental system that is a cornerstone of this study.

Fig.3A FACS plots for NCAM and CD44 are duplicated in supplemental Figure 4A, B . Authors should consider revising and perhaps making one common figure.

Referee #3:

This is a re-review. The authors adequately responded to the comments in the original review.

2nd Revision - authors' response 22 October 2012

We greatly appreciate your review and the meticulous referee's comments.

- The description of all reported data that includes statistical testing must state the name of the statistical test used to generate error bars and P values, the number (n) of independent experiments underlying each data point (not replicate measures of one sample), and the actual P value for each test (not merely 'significant' or 'P < 0.05').

Thank you for this clarification. In the current revised manuscript all statistical analyses include definite p values. We have inserted the names of statistical tests used to generate error bars and p values for each experiment. In addition, the number (n) of each independent experiment was indicated. (See Statistical analysis section in experimental procedures and figure legends).

- In addition, we noted some minor concerns regarding the figures: Please ensure that the resolution of the line graphs is improved and that labels are readable also at lower magnification.

According to the reviewer's comment, we have now improved the resolution of the line graphs in the manuscript.

- The section For More Information should be used to list relevant web links for further consultation by our readers. Examples include patient associations, relevant databases, OMIM/proteins/genes links or author's websites. Since you listed on the SI, please either remove the For More Information section or include relevant links as described above.

We have included relevant links for the described section.

<u>*Referee #2*</u>

The manuscript has been significantly improved after authors addressed previous concerns and performed additional experiments. This work is now suitable for publication with few minor remarks **:**

-Figure S1. (WT Xenograft system) should be considered as the main figure and be brought from supplemental section into the main text as it illustrates entire in-vivo experimental system that is a cornerstone of this study **.**

We thank the referee for the comment. We have decided not to include Fig S1 in the main test due to length constrains.

Fig.3A FACS plots for NCAM and CD44 are duplicated in supplemental Figure 4A, B. Authors should consider revising and perhaps making one common figure.

We are aware of the minor duplication in Fig 3A and Fig. S4B; we wish to keep Fig S4Aa and S4Ba as they are, in order to present all the details regarding CD44 and PSA-NCAM at one figure.

We hope that you will now find our revised manuscript suitable for publication in EMBO Molecular Medicine and we will be able to proceed with the official acceptance of the manuscript.
