# Supplementary material for: The isolation and characterization of renal cancer initiating cells from human Wilms' tumour xenografts unveils new therapeutic targets
Source: EMBO Mol Med. 2012 Dec 13;5(1):18–37. doi: 10.1002/emmm.201201516 (PMC3569651; doi:10.1002/emmm.201201516)
Supplement: Supplementary file 6 [file emmm0005-0018-SD6.pdf]

Supplemental information – Table of content:

SUPPLEMENTAL FIGURES AND LEGENDS **2**
FIGURE S1| WT XENOGRAFT SYSTEM 2
FIGURE S2| GROWTH CONDITIONS OF WT CELLS DETERMINE P-WT XN PHENOTYPE. 4
FIGURE S3| WT CANCER INITIATING AND PROPAGATING CELLS ARE CONTAINED WITHIN THE NCAM$^+$ POPULATION 6
FIGURE S4| ANALYSIS OF ADDITIONAL MARKERS WITHIN THE NCAM$^+$ CELL POPULATION REVEALS. 8
FIGURE S5| SORTING GATES AND PURITY OF FACS SORTING ACCORDING TO NCAM EXPRESSION AND ALDH1 ACTIVITY. 10
FIGURE S6| FIRST LINE CHEMOTHERAPIES DO NOT ELIMINATE NCAM$^+$ALDH1$^+$ CELLS. 12
FIGURE S7| EFFECT OF LORVOTUZUMAB MERTANSINE ON THE WT INITIATING CELLS IN VITRO 14
FIGURE S8| FACS ANALYSIS FOR THE PRESENCE OF NCAM$^+$ALDH1$^+$ (WT INITIATING CELLS) IN TWO RELAPSING TUMORS 16
SUPPLEMENTAL TABLES **17**
TABLE S1: PATIENT AND TUMOR CHARACTERISTICS 17
TABLE S2: OPTIMIZED CONDITIONS FOR SINGLE CELL TUMORIGENICITY IN IMMUNODEFICIENT MICE 18
TABLE S3: TUMORIGENICITY OF FRESH OR FROZEN/THAWED WILMS' TUMOR XENOGRAFT-DERIVED CELLS 18
LEGENDS FOR TABLES S4 AND S5 (DATASETS) **19**
TABLE S4: COMPARISON OF PROTEIN EXPRESSION BETWEEN UN-SORTED P-WT XN, NCAM$^+$ DERIVED P-WT XN, HUMAN FETAL AND ADULT KIDNEY TISSUES (PROVIDED AS XLS. DATASET FILE). 19
TABLE S5: MICROARRAY DATA OF DIFFERENTIALLY EXPRESSED MICRORNAS BETWEEN UN-SORTED P-WT XN, NCAM$^+$ALDH1$^+$ DERIVED P-WT XN AND HUMAN FETAL KIDNEYS (PROVIDED AS XLS. DATASET FILE). 19
LEGENDS FOR SUPPLEMENTAL MOVIES **20**
MOVIES S1 AND S2: NCAM$^+$ CELLS SHOW ENHANCE IN VITRO MOTILITY COMPARED TO NCAM$^-$ PRIMARY WT CELLS. 20
SUPPLEMENTAL EXPERIMENTAL PROCEDURES **21**
FLUORESCENCE-ACTIVATED CELL SORTING (FACS). 21
QUANTITATIVE REVERSE TRANSCRIPTION PCR ANALYSIS. 22
IMMUNOHISTOCHEMICAL STAINING OF PRIMARY WT AND WT XN. 22
KI67 IMMUNOSTAINING ANALYSIS (QUANTIFICATION). 22
NONDENATURING POLYACRYLAMIDE GEL ELECTROPHORESIS. 23
IMMUNOBLOT ANALYSIS. 23
COLONY FORMING ASSAY 23
PANORAMA® ANTIBODY ARRAY (SIGMA-ALDRICH, ST. LOUIS). 24
MICRORNA MICROARRAY - METHODS AND STATISTICAL ANALYSIS 24
TREATMENT OF WT CELLS WITH COMBINATION CHEMOTHERAPY. 25
IN VITRO EFFECTS OF CHEMOTHERAPEUTIC DRUGS ON WT INITIATING CELLS. 25
TREATMENT OF WT CELLS WITH LORVOTUZUMAB MERTANSINE IN VITRO. 26
ASSESSMENT OF WT CELL SURVIVAL. 26
ASSESSMENT OF WT CELL SURVIVAL AND NCAM EXPRESSION FOLLOWING TREATMENT WITH LORVOTUZUMAB MERTANSINE IN CORRELATION WITH INITIAL NCAM EXPRESSION. 27

Supplemental figures and legends

Fig. S1

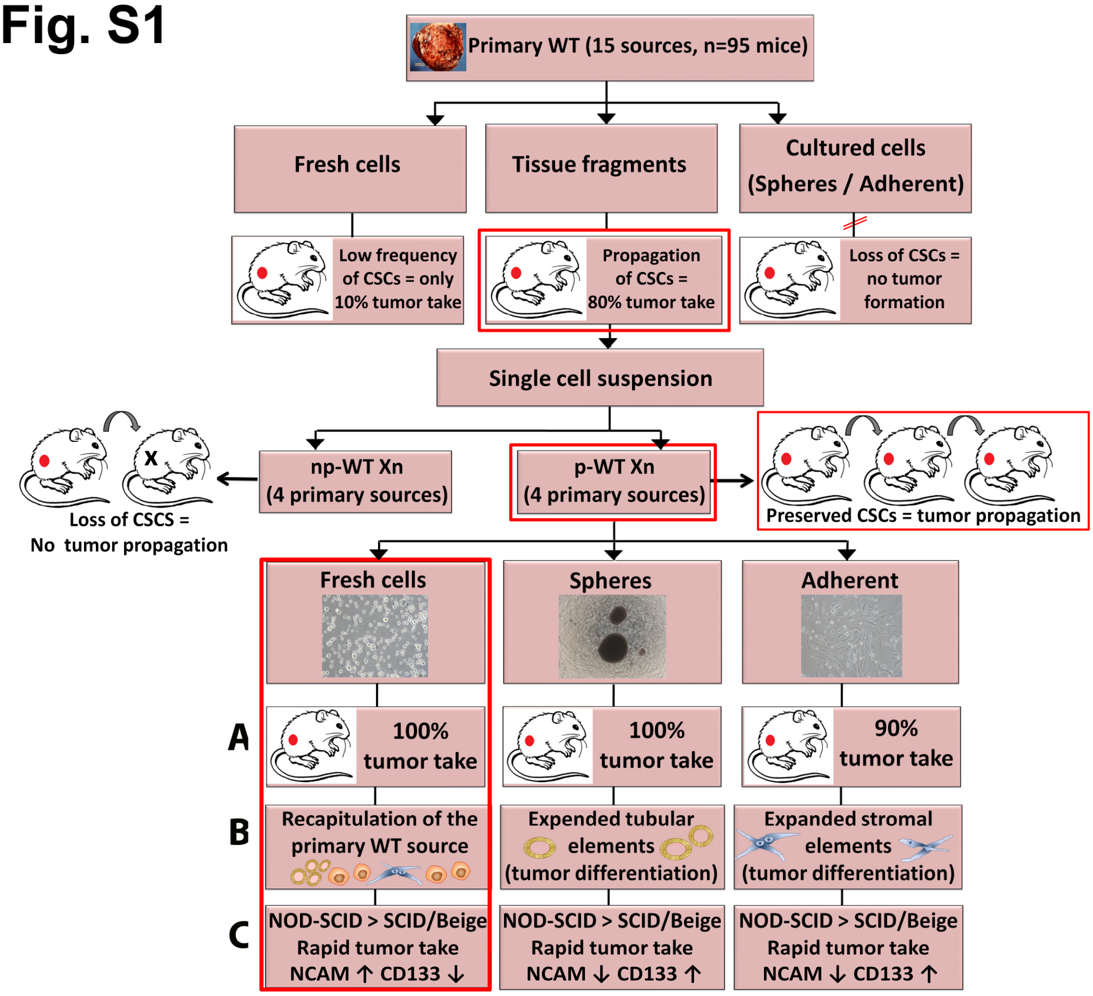

Figure S1| WT Xenograft system.

Experimental outline for WT Xn generation - scheme. WT xenografts are readily established from tissue fragment implantation (80% graft take) but are rarely or never formed by injection of single fresh primary WT cells (10% graft take) or cultured WT cells (0% graft take) – indicating loss of CIC/CSCs in primary cultures; the WT xenografts are then turned into a single cell suspension and tested for tumorigenic capacity. Two types of WT xenografts are formed: 1) propagatable WT xenografts (p-WT Xn) that can be serially and efficiently propagated in NOD-SCID mice via injection of single cells hence preserving putative CIC/CSCs, and 2) non propagatable WT xenografts (np-WT Xn) that are unable to establish xenografts in NOD-SCID mice via xenografts-derived single cell suspension indicating loss of the CIC/CSC; Different growth conditions alter p-WT Xn histology and *In vivo* tumor formation capacity; p-WT Xn derived cells injected into two strains of immunesuppressed mice

(NOD-SCID or SCID/Beige), either as fresh single cell suspension or after growth in low attachment conditions as spheres or as monolayer. (A) All conditions (fresh, spheres, adherent) could eventually initiate tumors in mice at similar frequencies; (B) All types of Xn-derived cells generated xenografts with tri-phasic components, however, tumors derived from fresh cells, while recapitulating the exacty histologic components seen in their parent tumor, contained increased amounts of undifferentiated blastema, while their counterparts initiated from spheres or adherent cells contained mostly differentiated structures: mature tubules were found in tumors derived from both spheres or adherent cells, while large areas of stroma were exclusively seen in those generated by adherent cells; (C) Lag time to tumor formation was shortest in NOD-SCID mice injected with fresh dissociated cells (see also table S3). Moreover, FACS analysis of cultured WT Xn-derived cells (P2-4) showed a reduction in NCAM expression and mostly the re-induction of CD133 in comparison to fresh Xn-derived cells (see also tables 1 and 2), coinciding with the ability of fresh cells to form more blastemal tumors and further suggestive of progenitor cell depletion in culture.

Fig. S2

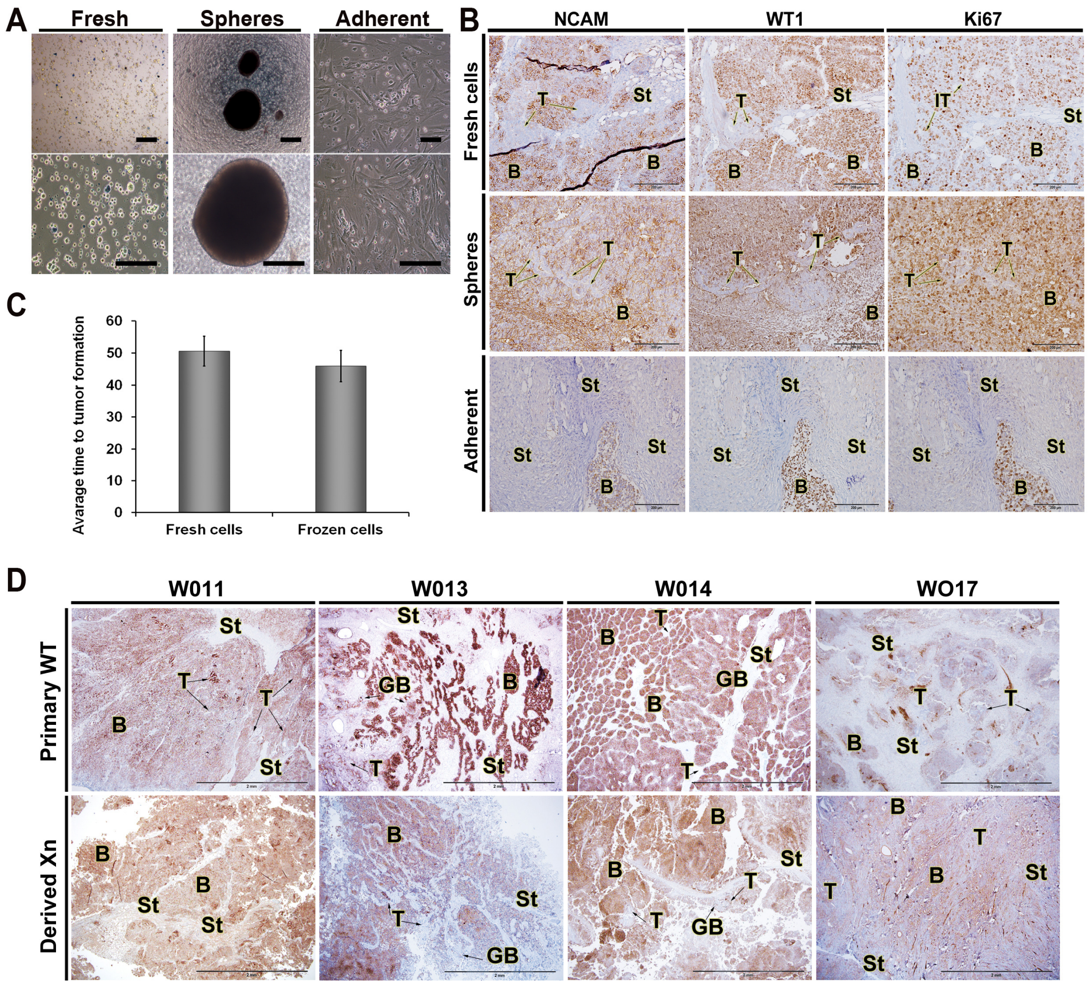

Figure S2| Growth conditions of WT cells determine p-WT Xn phenotype.

(A) Representative phase-contrast images of p-WT Xn derived cells injected into immunosupressed mice, either as fresh single cells suspension or grown in low attachment conditions as spheres or in adherent promoting conditions (Left panel, Scales bars=100μm, magnifications: 10x and 20x); (B) Immunohistochemical staining presented in serial sections for NCAM, WT1 and the proliferation marker Ki67 in WT Xn (W011) formed by each cell type (fresh, spheres and adherent). WT Xn derived from cultured cells (either spheres or adherent) demonstrates an abundance of differentiated structures (tubules and stroma) as opposed to an increase in the undifferentiated blastemal component in Xn derived from fresh single cell suspension. T-Tubules; B-Blastema; GB-Glomeruloid bodies; St-Stroma; IT-Immature Tubules. (Scales bars=200μm, magnification 20x); (C) The effect of freezing/thawing WT Xn cells on the time to tumor growth was measured by comparing the lag time in mice injected with fresh cells with those injected with frozen/thawed cells. No significant

influence of freezing/thawing on tumor growth was detected. All growth condition experiments were performed on 2 p-WT sources and 2 mice were used for each test group; (D) Immunohistochemical staining for NCAM expression presented as a panoramic view of representative Wilms' tumors included in this work with either a p-WT Xn (W011, W013, W014) or a np-WT Xn (W017) phenotypes. Tight histologic similarities can be seen between parental tumors and their derived p-WT Xns while a dramatic reduction in the amounts of NCAM expressing blastema can be observed in the np-WT Xn compared to its primary source. (Scales bars=2mm, Magnification x4).

Fig. S3

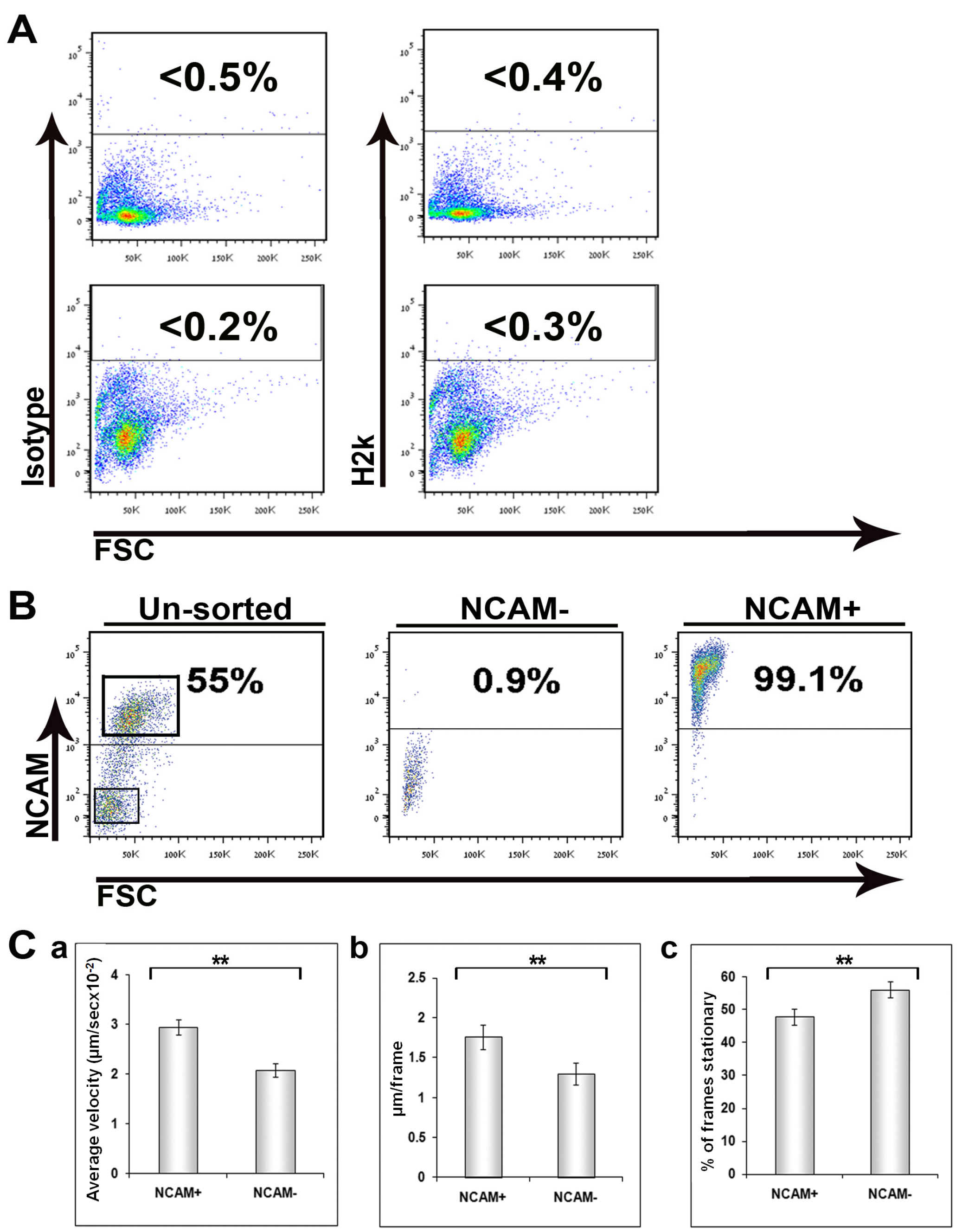

Figure S3| WT cancer initiating and propagating cells are contained within the NCAM+ population.

(A) Representative FACS analyses of H2k expression in WT Xn derived cells used for sorting. No H2k staining was seen in WT Xn suggesting lack of mouse tissue contamination; (B) Representative sorting gates used for sorting of p-WT Xn derived cells according to NCAM expression and the post sort analysis showing maximal sorting purity; (C) NCAM+ primary WT cells show greater in vitro motility than

$NCAM^-$ cells. Time-lapse movies of primary cultured WT cells FACS-separated by NCAM expression were made. Analysis shown is of 58 cells from 8 movies. (a) $NCAM^+$ WT cells moved faster (average velocity) than $NCAM^-$ WT cells. (b) When corrected for the number of frames, $NCAM^+$ WT cells also traveled greater distances than the $NCAM^-$ cells. (c) $NCAM^-$ cells were stationary more frequently than the $NCAM^+$ cells. **, $p<0.005$, ***, $p<0.001$. (See also supplemental movies S1 and S2).

Fig. S4

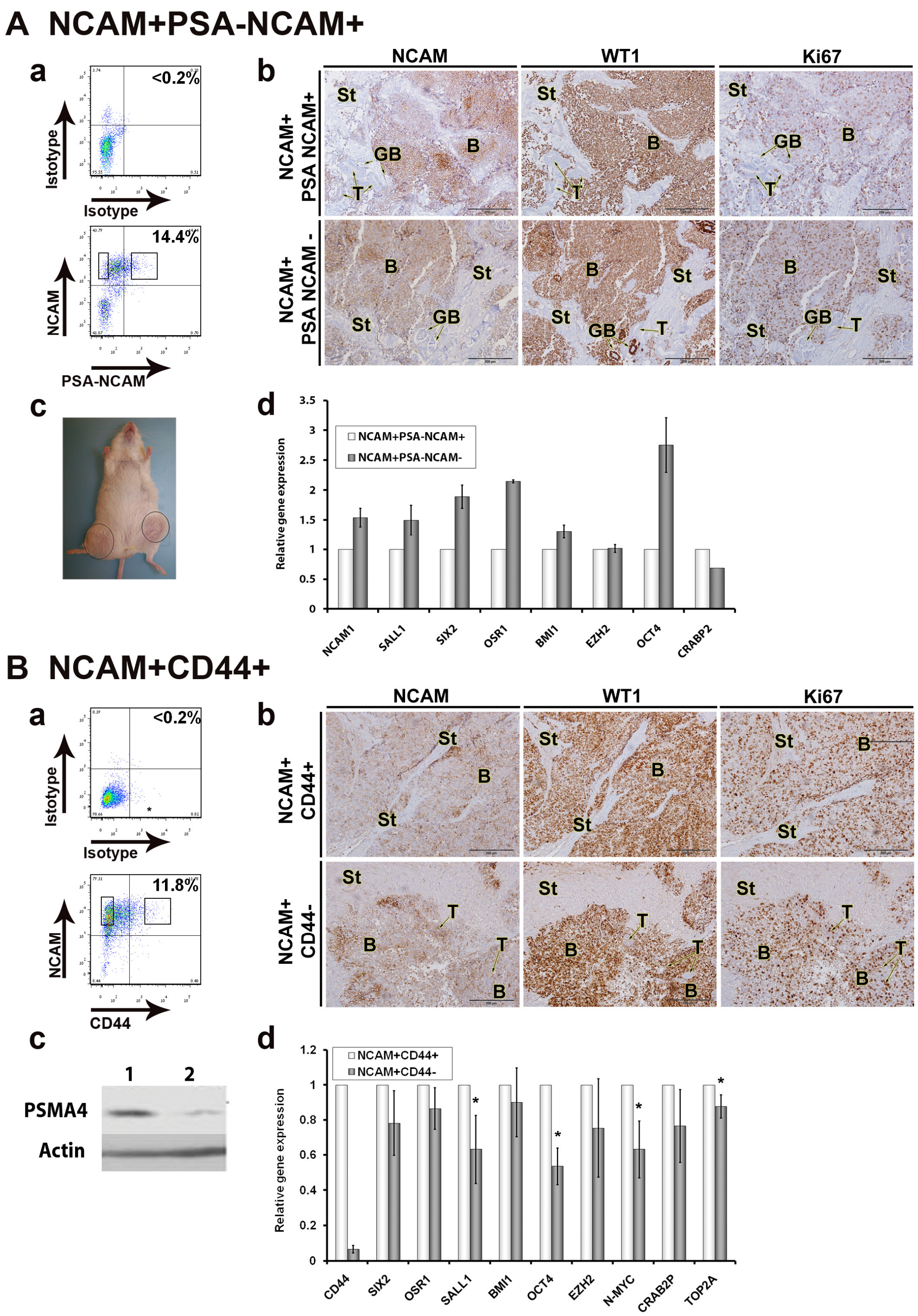

Figure S4| Analysis of additional markers within the NCAM^{+} cell population reveals.

(A) NCAM^{+}PSA-NCAM^{+}. (a) Representative FACS analysis showing PSA-NCAM to be expressed by cells within the NCAM^{+} population. (b) Immunohistochemical

staining presented in serial sections of tumors formed from either $NCAM^+$PSA-$NCAM^+$ or $NCAM^+$PSA-$NCAM^-$ cells for NCAM, WT1 and Ki67 demonstrate both tumors to be histologically similar (Scales bars=200µm; Magnification 20x). (c) A representative image of an NOD-SCID mouse injected with $NCAM^+$PSA-$NCAM^+$ (right flank) and $NCAM^+$PSA-$NCAM^-$ (left flank) cells that developed tumors from both subpopulations. (d) qRT-PCR analysis of renal progenitor, stemness and poor prognostic genes between $NCAM^+$PSA-$NCAM^+$ and $NCAM^+$PSA-$NCAM^-$ WT Xn cells, demonstrate similar mRNA levels of these genes in both cell populations. (B) $NCAM^+CD44^+$. (a) Representative FACS analysis showing CD44, to be expressed by cells within the $NCAM^+$ population. (b) Immunohistochemical staining of tumors formed from either $NCAM^+CD44^+$ or $NCAM^+CD44^-$ cells for NCAM, WT1 and Ki67 demonstrate both tumors to be histologically similar; (Scale bars=200µm; Magnification 20x). (c) Proteasomal levels were compared between WT Xn generated from the two cell fractions (1-W011 Xn $NCAM^+CD44^+$ and 2-W011 Xn $NCAM^+CD44^-$). Levels were higher in Xn derived from $NCAM^+CD44^+$ compared to $NCAM^+CD44^-$ cells; proteasomal subunit protein levels were analyzed by immunoblot analysis of polyacrylamide-SDS gel. Bar graph represents the relative band density of the upper image. (d) qRT-PCR analysis of renal progenitor, stemness and poor prognostic genes between $NCAM^+CD44^+$ and $NCAM^+CD44^-$ WT Xn cells, demonstrates higher mRNA levels of these genes in $NCAM^+CD44^+$, however only differences in SALL1, OCT4, N-MYC and TOP2A expression reached statistical significance (*, $p<0.031$).

Fig. S5

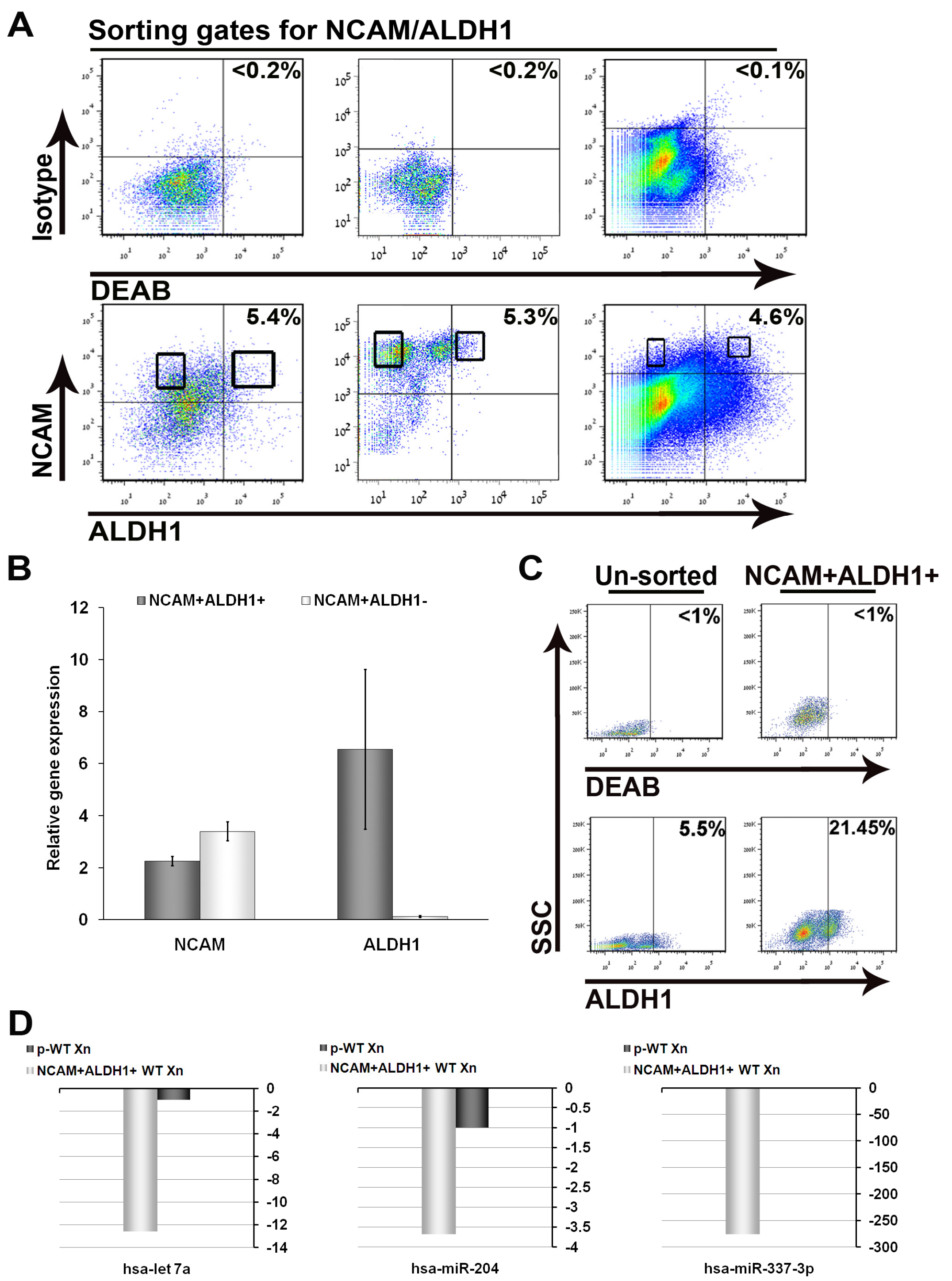

Figure S5| Sorting gates and purity of FACS sorting according to NCAM expression and ALDH1 activity.

(A) Representative sorting gates used for sorting of p-WT Xn according to NCAM expression and ALDH1 activity from three independent soring experiments of different WT Xn sources; (B) Analysis of NCAM and ALDH1 sorting purity via

qRT-PCR in the sorted fractions showing high ALDH1 expression in the $NCAM^{+}ALDH1^{+}$ cells and minimal to no expression in the $NCAM^{+}ALDH1^{-}$ cells while NCAM levels are comparable between the $NCAM^{+}ALDH1^{+}$ and $NCAM^{+}ALDH1^{-}$ populations; (C) Representative FACS plots of ALDH1 activity in p-WT Xn generated from unsorted cells and Xn generated from the same p-WT Xn (W014) sorted according to NCAM expression and ALDH1 activity ($NCAM^{+}ALDH1^{+}$ cells). Plots show an enrichment for cells with high ALDH1 activity in the sorted compared to unsorted xenografts as well as the appearance of cells lacking ALDH1 activity ($ALDH1^{-}$) that originate from $ALDH1^{+}$ cells; (D) qRT-PCR validation of three of the miRNAs (miR337, miR204 and let-7a) shown in the microaRNA microarray to be considerably downregualted in the $NCAM^{+}ALDH1^{+}$ derived p-WT Xn compared to un sorted WT Xn supporting the microarray results.

Fig. S6

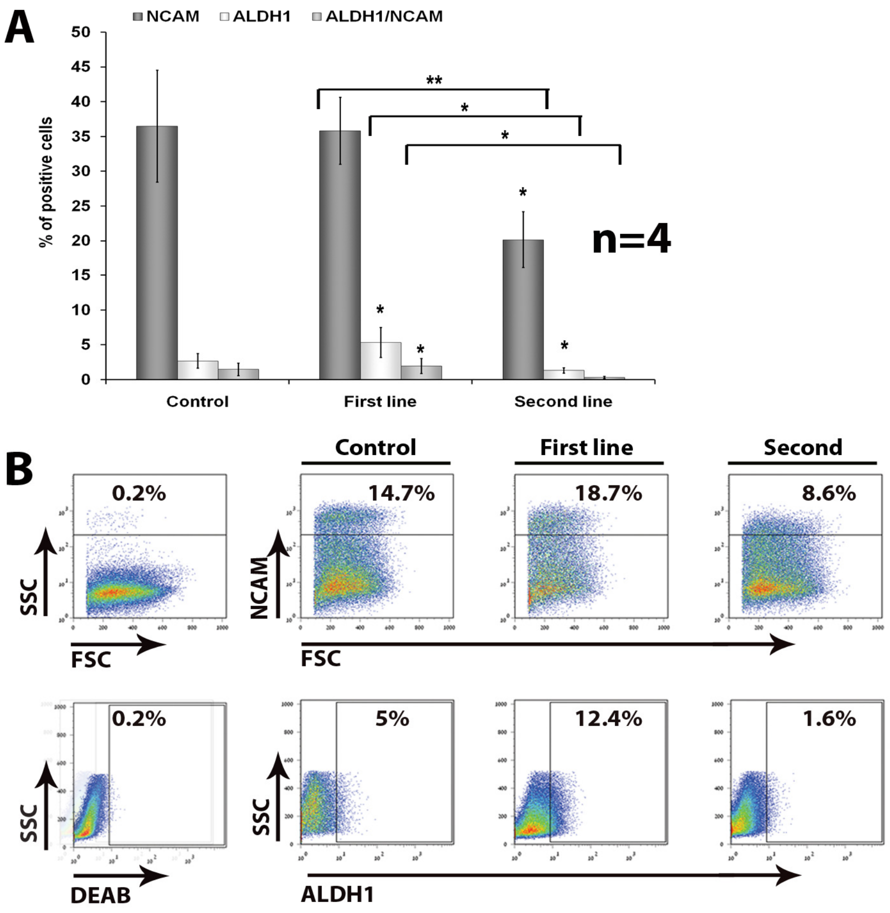

Figure S6| First line chemotherapies do not eliminate NCAM$^+$ALDH1$^+$ cells.

(A) The effects of the first line (vincristine and Actinomycin D) and the second line chemotherapeutic combination (etoposide and cisplatin) used in the clinic to treat WTs on the percentages of cells expressing NCAM (NCAM$^+$) or those with high ALDH1 activity (ALDH1$^+$) or both (NCAM$^+$ALDH1$^+$) were assessed by FACS. First line chemotherapeutic combination does not decrease the percentage of NCAM expressing cells and lead to a increase in the percentages of ALDH1$^+$ ($p=0.06$) and a significant increase in NCAM$^+$ALDH1$^+$ cells in comparison to untreated control (*, $p=0.05$). Application of the second line chemotherapeutic combination results in a significant decrease in the percentages of NCAM$^+$, ALDH1$^+$ and ALDH1$^+$NCAM$^+$ cells. Bar graph showing summation of experiments from 4 different primary WT sources (n=4). *, $p<0.05$, **, $p<0.01$ from control group, unless indicated otherwise. P values for the reduction in NCAM, ALDH1 and NCAM/ALDH1 by second line chemotherapy in comparison to untreated control are: 0.05, 0.049 and 0.07

respectively. P values for the reduction in NCAM, ALDH1 and NCAM/ALDH1 in by second line chemotherapy in comparison to first line chemotherapy are: 0.008, 0.049 and 0.05 respectively; (B) Representative FACS plots of WT cells treated either with first line or second line chemotherapeutic combination in comparison to untreated control, showing an increase in the percentages of $NCAM^{+}$ and $ALDH1^{+}$ cells in the first line treated WT cultures while decreased percentages of either cell type is observed in second line treated WT cells in comparison to untreated control.

Fig. S7

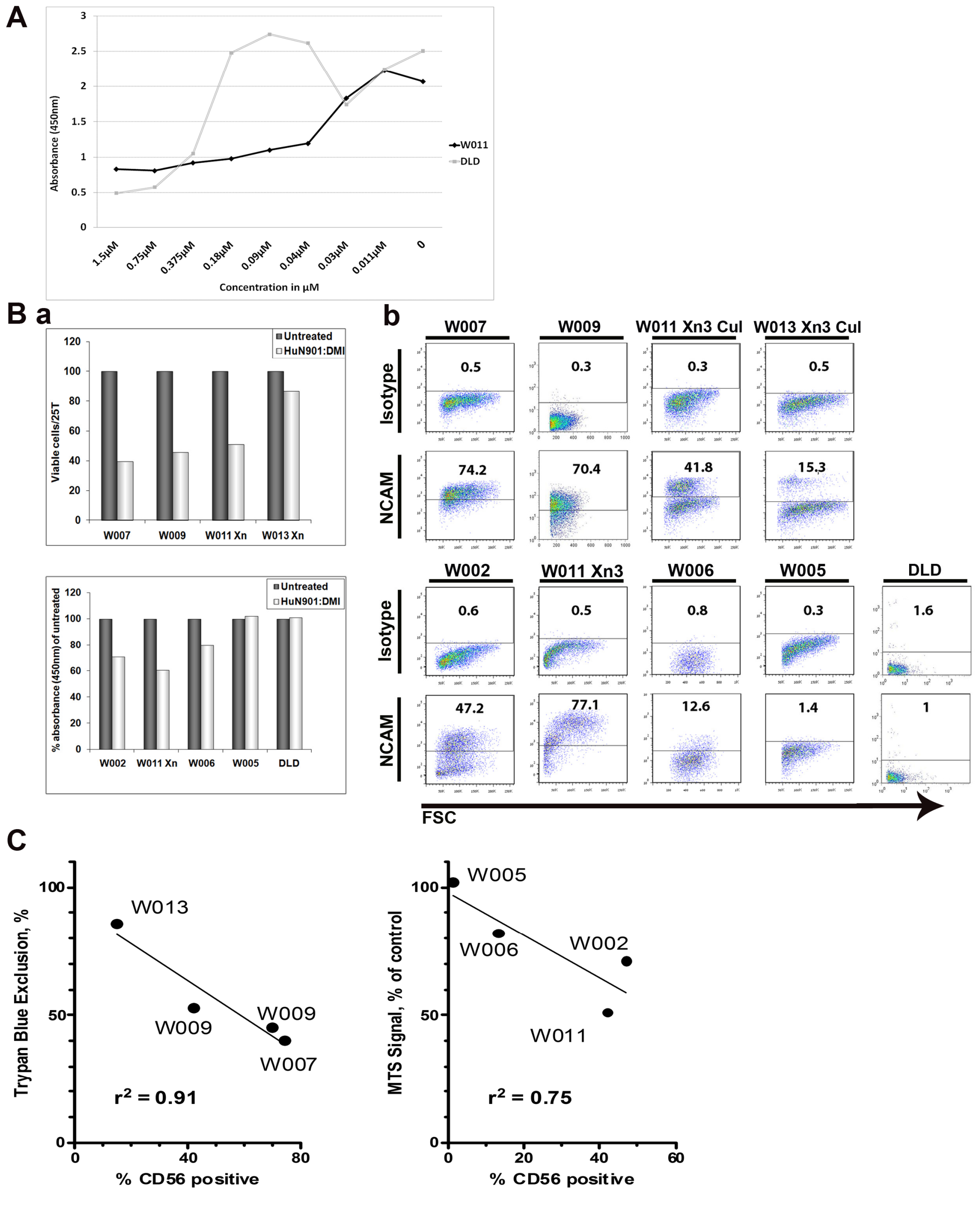

Figure S7| Effect of Lorvotuzumab mertansine on the WT initiating cells in vitro.

(A) MTS survival assay was used to determine the IC50 concentrations for lorvotuzumab mertansine on W011 WT Xn derived fresh cells (82% NCAM-positive) (black line) – IC50=0.046µM, and DLD cell line (NCAM negative) (gray line); Correlation between the percentage of NCAM-positive cells in a WT cell population and its sensitivity to the cytotoxic effect of lorvotuzumab mertansine (linear regression). (B) WT cell survival after treatment with lorvotuzumab mertansin. (a)

(Left) Trypan blue staining in untreated versus treated WT cells from 2 primary WTs (W007 and W009) and 2 cultured 3rd generation WT Xn (W011 Xn3 Cul and W013 Xn3 Cul). (Lower panel) MTS proliferation assay performed on treated and untreated cells from 4 different patients (three primary cultured tumors – W006, W007 and W005 and one freshly dissociated 3rd generation WT Xn – W011 Xn3). Data are presented as the percentage of absorbance at 450nm of the treated cells from that of the untreated control for each tumor; DLD cell line was used as a negative control. Experiments were repeated twice in triplicates. (b) (Right) FACS analysis demonstrating a distinct correlation between the percentage of dead cells and the percentage of NCAM expressing cells in each of the tumors analyzed was observed (see also figure 30); (C) Linear regression was used in order to show the correlation between the percentage of NCAM expressing cells in different tumors with the percentage of cell death (Trypan blue exclusion assay) or cell proliferation (MTS assay) following treatment with lorvotuzumab mertansine.

Fig. S8

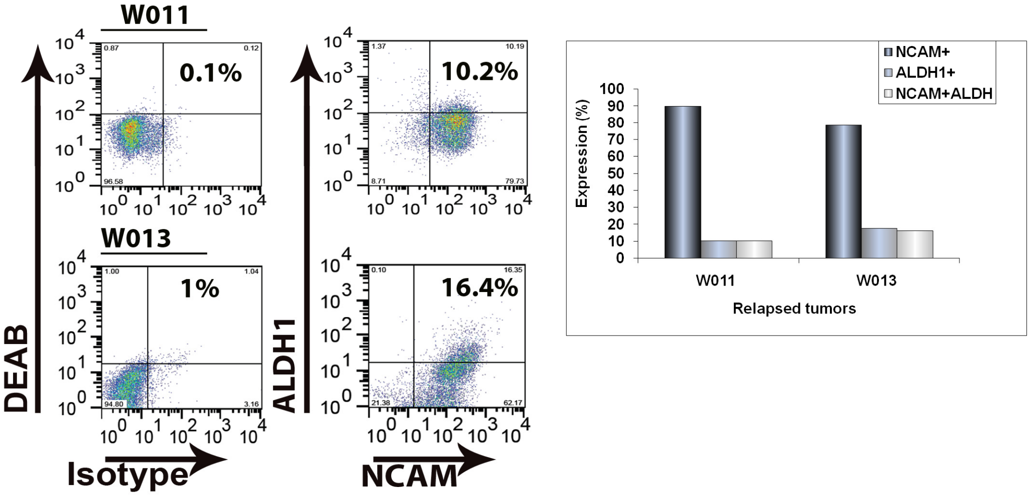

Figure S8| FACS analysis for the presence of NCAM^{+}ALDH1^{+} (WT initiating cells) in two relapsing tumors

FACS analysis for the presence of NCAM^{+}ALDH1^{+} in two relapsing tumors (post treatment with *lorvotuzumab mertansine)*, showed a significant and steady NCAM^{+}ALDH1^{+} population in both tumors.

Supplemental tables

Table S1: Patient and tumor characteristics

Patient Code	Gender	Age	Pattern	Histology	Remarks
W002	Female	4 years	Triphasic	Favorable	Lung metastasis
W003	Male	10 years	Triphasic - Blastemal predominance	Unfavorable	Recurrent with diffused anaplasia
W004	Female	6 years	Triphasic	Favorable	Bilateral
W005	Male	3 years	Triphasic	Favorable	-
W006	Male	2 years	Triphasic	Favorable	Focal anaplasia
W007	Female	3 years	Triphasic	Favorable	Recurrent WT with focal anaplasia
W009	Male	3 years	Triphasic	Unfavorable	Recurrent with diffused anaplasia
W010	Female	1 years	Triphasic	Favorable	-
W011	Female	7 years	Triphasic - Blastemal predominance	Favorable	Recurrent WT with Liver metastasis
W013	Male	4 years	Triphasic	Favorable	-
W014	Male	9 months	Triphasic	Favorable	-
W016	Female	2 rears	Triphasic	Favorable	-
W017	Male	3 years	Triphasic	Favorable	-
W019	Male	1 years	Biphasic – mostly stromal and blastemal	Favorable	-

Table S2: Optimized conditions for single cell tumorigenicity in immunodeficient mice

Weeks post injection	*NOD-SCID*			*SCID/BG*		
	Fresh	***Spheres***	***Adherent***	***Fresh***	***Spheres***	***Adherent***
1 week	**1/3**	0/3	0/3	0/3	0/3	0/3
2 weeks	**1/3**	1/3	0/3	0/3	0/3	0/3
4 weeks	**3/3**	2/3	2/3	2/3	1/3	2/3
6 weeks	**3/3**	3/3	3/3	3/3	3/3	3/3

Table S3: Tumorigenicity of fresh or frozen/thawed Wilms' tumor xenograft-derived cells

Xn Code	***Cells***	***1 Week***	***2 Week***	***3 Week***	***4 Week***	***5 Week***	***6 Week***	***7 Week***	***8 Week***
W011	**Fresh**	0/2	0/2	0/2	0/2	0/2	1/2	2/2	2/2
	**Frozen**	0/2	0/2	0/2	0/2	1/2	2/2	2/2	2/2
W013	**Fresh**	0/2	0/2	0/2	0/2	0/2	2/2	2/2	2/2
	**Frozen**	0/2	0/2	0/2	0/2	0/2	2/2	2/2	2/2
W014	**Fresh**	0/2	0/2	0/2	1/2	1/2	1/2	1/2	1/2
	**Frozen**	0/2	0/2	0/2	0/2	0/2	2/2	2/2	2/2

Legends for tables S4 and S5 (Datasets)

Table S4: Comparison of protein expression between un-sorted p-WT Xn, NCAM$^+$ derived p-WT Xn, human fetal and adult kidney tissues (provided as xls. Dataset file).

Panorama antibody array was used (725 validated antibodies that detect proteins associated with a variety of key cell signaling and gene regulation pathways) on extracts of p-WT Xns early after initiation by sorted NCAM$^+$ cells, un-sorted p-WT Xn and normal kidneys. We considered differentially expressed proteins only when at least a five-fold change appeared among samples. Proteins that were preferentially activated in NCAM$^+$ derived tumors were confirmed by Western blot (figure 3E). Of signaling molecules specific up-regulation of Ser473-phosphorylated Akt (AKTpSer473) was detected in NCAM$^+$ p-WT Xn.

Table S5: Microarray data of differentially expressed microRNAs between un-sorted p-WT Xn, NCAM$^+$ALDH1$^+$ derived p-WT Xn and human fetal kidneys (provided as xls. Dataset file).

71 microRNAs were differentially expressed between human FKs and p-WT Xn tissues. Expression pattern was similar among all p-WT Xns, with 35 down-regulated and 36 up-regulated microRNAs (miRs) compared to the hFK tissues. The most significantly reduced microRNAs were microRNA families: let7 and 200 and miR-204. Changes in miR expression were more accentuated in NCAM$^+$ALDH1$^+$ derived WT Xn compared to unsorted p-WT Xns. microRNA family let-7, miR-200a and miR-200b showed approximately 2- fold and miR-204 8- fold reduction in NCAM$^+$ALDH1$^+$ derived WT Xn compared to unsorted p-WT Xn and a larger reduction when compared to human fetal kidney (microRNA family let-7: let-7a 4-fold, let-7d 5- fold, let-7f 5- fold, let-7g 6- fold; microRNA family 200: miR-200a 94-fold, miR-200b 75- fold, miR-200c 29- fold, miR-141 34- fold, miR-429 54- fold and microRNA 204 226- fold reduction).

Legends for supplemental movies

Movies S1 and S2: $NCAM^+$ cells show enhance in vitro motility compared to $NCAM^-$ primary WT cells.

Representative time lap-video microscopy movies of primary cultured, FACS-sorted Wilms' tumor $NCAM^+$ cells and $NCAM^-$ cells showing enhance in vitro motility of the former. Supplemental figure S3B shows analysis of motility characteristics of 58 cells from 8 movies of $NCAM^+$ and $NCAM^-$ cells.

Supplemental experimental procedures

Fluorescence-activated cell sorting (FACS).

FACS analysis of both fresh and cultured WT cells was performed as previously described. Cells grown under adherent conditions were harvested using 0.05% Trypsin/EDTA (Gibco, Grand Island, NY). Cells grown as spheres were dissociated by trypsinization for 1min at 37°C. Detection of cells with high ALDH1 enzymatic activity was performed using the ALDEFLUOR kit (StemCell Technologies, Durham, NC, USA) as previously described (Christophe Ginestier et al., 2007; Dylla et al., 2008). As negative control, for each sample of cells an aliquot was treated with DEAB, a specific ALDH inhibitor. For double staining selection of ALDH1 activity and NCAM expression, cells were suspended in ALDEFLUOR buffer and the fluorescent anti-NCAM antibody was added followed by 45min incubation in the dark at 4°C and washing steps as described above. 7-amino-actinomycin-D (7AAD; eBioscience, San Diego, CA) was used for viable cell gating and quadrants were placed according to both the DEAB and the isotype-matched APC-labeled non-targeting antibody as a control for the APC-labeled anti–NCAM MEM188 antibody (eBioscience, San Diego, CA). For cell sorting, small pieces of WT Xn tumors were dissociated into single cells, washed, RBCs lysed, filtered through a 30µm nylon mesh before final centrifugation, then resuspended in flow cytometry buffer (0.1% bovine serum albumin (BSA; Sigma-Aldrich, St. Louis) in PBS). Cells were labeled with anti-NCAM:APC alone or in combination with anti-CD44:PE or anti-PSA-NCAM:PE antibodies and remaining steps were as described above. For separation of cells with high ALDH activity, single cell suspension of WT Xn was re suspended in ALDEFLUOR assay buffer containing ALDH substrate and treated as previously described (Christophe Ginestier et al., 2007; Dylla et al., 2008). Possible contaminating mouse cells were eliminated by discarding $H2K^+$ (with an anti-H2Kd antibody - Miltenyi Biotech, Germany), $CD31^+$ and $CD45^+$ (with an anti-CD31:FITC and anti-CD45:FITC antibodies - San Diego, CA) mouse cells. Fluorescence-activated cell sorter FACSAria (BD Biosciences, San Jose, CA) was used in order to enrich for cells expressing these markers. Data was additionally analyzed and presented using FlowJo software (Tree Star, Ashland, OR).

Quantitative reverse transcription PCR analysis.

Quantitative reverse transcription PCR (qRT-PCR), to determine expression of renal progenitor (*SIX2, OSR1, SALL1*), stemness (*BMI1, EZH2, OCT4*) and poor prognosis (*TOP2A, N-MYC, CRAB2P*) genes in primary WT, WT Xn and sorted WT Xn cell subpopulations was carried out as previously described (Dekel et al., 2008). RNA was extracted using the microRNeasy kit (Qiagen) and cDNA synthesis was carried out using the High capacity cDNA RT kit (Applied Biosystems, Foster City, California, USA) according to the manufacturer's instructions. Each reaction was performed in duplicate. *HPRT1* or *GAPDH* were used as endogenous controls. Gene expression analysis was performed using TaqMan Gene Expression Assays on an ABI Prism 7900HT sequence detection system (Applied Biosystems, Foster City, California, USA).

Immunohistochemical staining of primary WT and WT Xn.

Sections, 4-µm thick, were cut from primary WT and WT Xn for immunohistochemistry. Immunostainings were performed as previously described (Dekel et al., 2006b). Brifly, the sections were processed within 1 week to avoid oxidation of antigens. Before immunostaining, sections were treated with 10mM citrate buffer, PH 6.0 for 10 min at 97ºC in a microwave oven for antigen retrieval, followed by 3% H_2O_2 for 10 min. The slides were subsequently stained using the labeled strepavidin-biotin (LAB-SA) method using a Histostain plus kit (Zymed, San Francisco, CA, USA). Anti human NCAM antibody (LifeSpan Biosciences, Inc. Seattle, WA, USA), anti human WT1 antibody and anti human Ki67 antibody, at a dilution of 1:50 were used. Controls were prepared by omitting the primary antibodies or by substituting goat IgG isotype for the primary antibodies. The immunoreaction was visualized by an HRP-based chromogen/substrate system (liquid DAB substrate kit – Zymed, San Francisco, CA, USA).

Ki67 immunostaining Analysis (Quantification).

Quantification of Ki67+ cells in blastema and non-blastemal components of two primary WTs (W013, W014) and their corresponding WT Xn (W013 Xn, W014 Xn) was performed as previously described (Ghanem, Van der Kwast et al, 2004). The slides were evaluated by two independent observers, using a standard light microscope with a ×60 objective and equipped with an ocular grid. Cells were

considered positive regardless of the intensity or location of nuclear staining. Quantification was performed by counting at least 1000 tumor cells in five randomly selected fields of view.

Nondenaturing polyacrylamide gel electrophoresis.

Cells were collected and homogenized in buffer containing 20 mM Tris-HCl(pH 7.5), 1 mM EDTA, 1 mM DTT, and 250 mM sucrose. The extract was centrifuged at 13000xg for 15 min. The supernatant subjected to ultracentrifugation for 16 hours at 100,000xg. The pellet was resuspended and loaded on a nondenaturing polyacrylamide gel using the protocol previously described (Tsvetkov et al., 2009).

Immunoblot analysis.

The protein mix was suspended in Laemmli buffer [4% SDS, 20% glycerol, 10% 2-mercaptoethanol and 0.125M Tris-HCl], heated at 95^0C for 5 minutes and loaded on a 10% polyacrylamide-SDS gel. Following electrophoresis, proteins were transferred to cellulose nitrate 0.45 mm membranes (Schleicher & Schuell, USA). The antibodies used were: Rabbit anti PSMA4 (provided by Prof. Kahana, WIS), PSMD1 (Acris), PCNA, Actin (Santa-Cruz, USA), PKBpSer473 (Sigma), NFkB (Santa-Cruz), and alpha tubulin (Sigma), anti-p53 (1801), anti Hsc70 (kindly provided by Dr. Evan Elliot), anti p-ERK (kindly provided by Dr. Rony Seger), anti-b-catenin (6F9 Sigma), anti-NQO1 (A180), anti-GAPDH, anti-c-Fos (H-125), anti-c-Jun (H-79), anti-YAP1 H125, anti-Src, anti-p-RSK and anti-R2 N-18 (Santa-cruz). Secondary antibodies were HRP-linked Goat anti-mouse and anti-rabbit (Jackson ImmunoResearch, USA). Signals were detected using the Ez-ECL kit (Biological Industries, Israel).

Colony forming assay.

Cells were routinely cultured in IMEM medium supplemented with 10% FBS ("growth medium"). For assessment of colony forming ability (CFU), primary WT cells before and after treatment with lorvotuzumab mertansine, or $NCAM^+ALDH1^+$ and $ALDH1^-$ sorted cells were plated in growth medium on matrigel-coated either 6 or 24 well plates at 1000 or 5000 cells/well in triplicate, respectively. The medium was changed twice a week. After two weeks both the number of colonies and the number of cells/colonies were determined, and means calculated.

Panorama® Antibody Array (Sigma-Aldrich, St. Louis).

Two frozen WT xenograft tissues from different sources were tested while AK and FK were used as comparative controls. The experiment was conducted according to the manufacturer's instructions: the tissues were homogenized on ice using a homogenizer and the proteins concentration were determined by the Bradford protein assay (Thermo scientific). Equal amounts of protein extracts (>1 mg/ml) were labeled using Cy3 monoreactive reactive dyes (Amersham Biosciences) as described by the manufacturer (Sigma-Aldrich, St. Louis). Labeled samples with a dye/protein molar ratio >2 were applied to the antibody microarray in Array Incubation Buffer (Sigma-Aldrich, St. Louis) and incubated for 45 min protected from light with gentle shaking. The array was then washed three times with 5 ml of Washing Buffer (Sigma-Aldrich, St. Louis), air-dried completely, and scanned using a GenePix 4000B microarray scanner (Axon Instruments, Foster City, CA). Image analysis was performed with GenePix Pro 5.0 software that provides a tool to obtain the position and content of each spot on the array.

microRNA Microarray - Methods and statistical analysis

Total RNA from tissues was isolated with a miRNeasy mini kit (Qiagen, Valencia, CA) using the manufacturer's protocol. MicroRNA profiling was performed with an 8 x 60K Agilent human microRNA microarray containing 1205 human and 144 human viral microRNAs (Sanger miRbase release 16.0), in accordance with the protocol described by the manufacturer (Agilent, Santa Clara, CA). (This information was adapted from Panditet al. paper described below.)

Statistical Analysis: MicroRNA microarray data was analyzed using the Genespring GX 11.0 software (Agilent, Santa Clara, CA). Expression values were log2 transformed and normalized using a Quantile Normalization method. Baseline transformation to the median expression of the controls [human fetal kidneys (hFK)] was performed and a One-Way ANOVA test with Benjamini-Hochberg multiple testing correction was used to identify those microRNAs (miRNAs) that were differentially expressed (p-value <0.05) between Wilms' Tumor Xenografs (p-WT Xn) and $NCAM^{+}ALDH^{+}$ derived WT Xn and control tissues (hFK); 4 different sources was used for each tissue type. Each miRNA has approximately 40 unique probes on the array. miRNAs which were unexpressed across all samples were

excluded prior to statistical analysis. Data visualization was accomplished using the Genespring GX 11.0 software (Pandit et al.).

The microarray data from this publication have been submitted to the GEO database [http://www.ncbi.nlm.nih.gov/geo/query/acc.cgi?acc=GSE33332]. The GEO Submission ID is GSE33332 and the NCBI tracking system number is 16192630.

Treatment of WT cells with combination chemotherapy.

In order to determine the in-vitro IC50 of WT cells for each combination of the studied drugs (first line – vincristine and actinomycin D and second line – etoposide and cisplatin), cells were seeded in 96 well plates at 10^4 cells/well for 48h. The medium was then replaced with medium containing a range of concentrations of both drug combinations: for vincristine and actinomycin D - 250µM-1µM and for etoposide and cisplatin - 250µM-1µM were tested for each of the drugs in the combination, medium without drugs served as the control. Following 48 h exposure, the MTS proliferation assay was performed in accordance with the manufacturer's instructions. The IC50 values of the vincristine/actinomycin D combination, and of the etoposide/cisplatin combination for W004 (primary WT) and W011 (WT Xn), were found to be 0.24µM and 62µM, respectively. All further experiments evaluating the effects of these drugs on WT cells were performed at these concentrations.

In vitro effects of chemotherapeutic drugs on WT initiating cells.

In order to examine the effect of the chemotherapy regimens on the percentage of cells expressing NCAM and ALDH1, WT cells (from each source) were plated in 3x75T flasks for 72h. Following the indicated time, medium was removed and replaced by medium containing the first line drug combination for the first flask, the second line drug combination for the second flask and no drugs for the third flask. The untreated flask was used as the baseline for NCAM and ALDH1 expression in each tumor examined. After treatment, cells were incubated for 48h, the medium was removed, cells were harvested using 0.05% Trypsin/EDTA, counted and analyzed by FACS for the percentage of cells expressing NCAM and ALDH1 as described above. To study the *in vitro* effect of the two chemotherapeutic combinations on the CIC fraction we assessed the CFU capacity (as described above) of the cells after treatment in comparison to untreated control.

Treatment of WT cells with lorvotuzumab mertansine in vitro.

Lorvotuzumab mertansine (huN901-DM1, formerly IMGN901), a humanized version of the anti-CD56 antibody N901 conjugated to the highly cytotoxic maytansine derivative DM1 via a hindered disulfide linker (Wang et al., 2005) was produced at ImmunoGen, Inc., Waltham, Massachusetts. In order to determine the IC50 of lorvotuzumab mertansine for WT cells, cells were seeded in 96 well plates at 10^4 cells/well for 24h in growth medium. The medium was then replaced with medium containing a range of concentrations of the conjugate between 0.1nM to 1.5μM, with medium alone serving as control. Following a five-day incubation, cell survival was assessed by the addition of (3-[4,5-dimethylthiazol-2-yl-5]-[3-carboxymethyoxyphenyl]-2-[4-sulfophenyl]-2H tetrazolium) MTS (Promega, Madison, WI) for 4h, followed by measuring absorbance at 450 nm on a microplate spectrophotometer in accordance with the manufacturer's instructions.

The IC50 value of lorvotuzumab mertansine for W011 WT Xn-derived fresh cells (from 3rd generation Xn=W011 Xn3, that express 82% NCAM) was 0.046μM. In order to ensure effective killing of close to a 100% of NCAM expressing cells (and not 50% as for the IC50 concentration) we used a concentration of 0.18μM that showed the most precise correlation between cell death after treatment (as assessed by trypan blue exclusion assay and MTS proliferation assay) and NCAM expression prior to treatment as determined by FACS analysis. All further experiments evaluating the effect of this drug on WT cells *in vitro* were performed at this concentration.

Assessment of WT cell survival.

To assess the effect of lorvotuzumab mertansine on WT cell survival in comparison to the unconjugated mAb, WT cells from 3 WT Xn derived from distinct patients (W016 Xn, W027 Xn and W028 Xn) were seeded in 96 well plates in growth medium at 10^4 cells/well. 24 h later medium was changed with growth medium either containing 0.18 μM of lorvotuzumab mertansine or containing 1μM of unconjugated mAb (HuN-901) or with regular growth medium (control) in triplicates. Following 5-day exposure, cell survival was assessed by the MTS proliferation assay as described above.

Assessment of WT cell survival and NCAM expression following treatment with lorvotuzumab mertansine in correlation with initial NCAM expression.

Two assays were used to assess WT cell survival after treatment with the conjugate: MTS proliferation assay and trypan blue exclusion assay. For the MTS assay, cells from four WTs from distinct patients (primary tumors: W005, W006, W007 and freshly dissociated 3rd generation W011 Xn cells: W011 Xn3) with different NCAM expression levels, as assessed by FACS prior to treatment, and grown, treated and assessed as described above. For the trypan blue exclusion assay, cultured cells from two primary WTs (W007 and W009) and two 3rd generation WT xenografts (W011 Xn3 Cul and W013 Xn3 Cul) were seeded in 25T flasks at 10^5 cells/flask and treated with lorvotuzumab mertansine or with medium alone as described for the MTS assay. Following 5 day-exposure, cells were harvested using 0.05% Trypsin/EDTA (Gibco, Grand Island, NY), and viable cells were counted with trypan blue as previously described (Songyang et al., 1997). Assessment of NCAM expression was performed on both treated and untreated cells by FACS analysis as described above.
